# SCT-YOLO: A Dual-Stream Defect Detection Network Utilizing Computational Shape, Texture, and Color Features

**DOI:** 10.3390/s26123662

**Published:** 2026-06-08

**Authors:** Zhenning Mou, Yuchao Dai, Zihe Cao, Zhe Lv, Lei Wang, Yehu Shen, Guizhong Fu

**Affiliations:** 1 School of Mechanical Engineering, Suzhou University of Science and Technology, No. 55, Changjiang Road, Suzhou 215009, China; 2311171011@post.usts.edu.cn (Z.M.); 2413171002@post.usts.edu.cn (Y.D.); leiwang@usts.edu.cn (L.W.); yehushen@mail.usts.edu.cn (Y.S.); 2 School of Advanced Technology, Xi’an Jiaotong-Liverpool University, No. 111, Ren’ai Road, Suzhou 215123, China; zihe.cao23@student.xjtlu.edu.cn; 3 School of Electronic & Information Engineering, Suzhou University of Science and Technology, No. 99, Xuefu Road, Suzhou 215009, China; jay.zhe.lu@gmail.com

**Keywords:** dual-stream architecture, feature fusion, attention mechanism, low-level visual features, industrial surface defect

## Abstract

Steel surface defect detection is a key component of industrial quality control. Existing deep learning methods mostly rely on single-backbone networks to extract high-level semantic features from raw images, yet fail to explicitly analyze low-level visual features. To overcome the limitations of conventional frameworks, this paper proposes SCT-YOLO, a defect detection model based on a dual-stream collaborative architecture that effectively integrates low-level visual features with high-level semantic information for complementary feature representation. The primary scientific novelty of this work lies in the formalization of a multi-dimensional prior feature-guided learning paradigm, which mathematically bridges explicit hand-crafted physical priors with deep latent representations within this dual-stream architecture, differing from conventional black-box deep feature extraction. On the SD-Saliency-900 dataset, the proposed SCT-YOLO achieves an mAP@0.5 of 88.9%, representing a significant 4.6% improvement over the baseline model YOLOv8n, while maintaining an inference speed of 257.2 FPS with only 5.66 M parameters and 15.2 GFLOPs, fully meeting the real-time deployment requirements of industrial production lines. Visualization analysis demonstrates that the method exhibits more stable detection capability for small defects in complex backgrounds. Meanwhile, experiments on the GC10-DET dataset further verify its excellent generalization performance, providing a reliable technical solution for other industrial defect detection scenarios.

## 1. Introduction

Steel, as a core fundamental material in industrial production, is widely applied in key fields such as infrastructure construction, automotive manufacturing and aerospace, and its surface quality directly determines the reliability, safety and service life of products [1,2,3]. During multi-stage production processes including steel rolling, heat treatment, processing and transportation, various surface defects such as cracks, scratches, rust spots and holes are prone to occur. If these defects are not detected in a timely manner, they may give rise to serious potential safety hazards such as structural failure and equipment malfunction [4,5]. Therefore, achieving high-precision and high-efficiency automatic detection of steel surface defects is of great significance for ensuring product quality, improving production efficiency and reducing maintenance costs.

Traditional steel surface defect detection mainly depends on manual visual inspection and methods based on simple photoelectric technology. These methods have such problems as low efficiency, strong subjectivity and poor consistency, making it difficult to meet the quality control requirements of modern large-scale industrial production [6]. With the development of machine vision technology, automatic detection methods based on image processing have gradually become a research hotspot. Early automatic detection methods mainly relied on manually designed feature extractors, such as edge detection, threshold segmentation, morphological operations and texture analysis [7,8]. However, these methods are heavily dependent on expert experience, sensitive to image resolution, illumination conditions and changes in defect morphology, and have limited generalization ability, which makes it difficult for them to adapt to changes in product batches, surface treatment processes and imaging conditions in actual industrial environments.

In recent years, the rapid development of deep learning technology has provided new solutions for industrial defect detection. Owing to their ability to automatically learn hierarchical representations, CNNs have gradually become the mainstream approach for surface defect detection. Compared with traditional methods, deep learning extracts multi-scale and semantically rich discriminative features directly from raw pixels through end-to-end learning without the need for manually designed features, which significantly improves the adaptability and accuracy of detection [9,10]. However, existing methods still face multiple challenges in industrial defect detection scenarios: the models are insufficient at capturing features of multi-scale defects, which is likely to lead to missed detections of tiny defects; there is a wide variety of defect types with low contrast against the complex background, which tends to cause false detections in actual detection tasks; industrial defect detection scenarios impose stringent requirements on detection stability and accuracy, yet existing methods still struggle to achieve reliable defect identification under complex industrial conditions.

Most existing deep learning methods directly perform end-to-end learning on raw RGB images without explicitly integrating prior visual feature knowledge that is crucial for specific tasks, which to a certain extent limits the perception accuracy and robustness of models in complex industrial scenarios. To address the above problems, this paper proposes a steel surface defect detection model named SCT-YOLO based on a dual-stream collaborative architecture.

To address these challenges, this study introduces a dual-stream collaborative detection network named SCT-YOLO. By incorporating a dual-backbone framework, the network parallelly processes raw images and multi-dimensional prior feature maps of shape, color, and texture, achieving an adaptive feature fusion that helps alleviate certain limitations of traditional single-backbone architectures. To integrate prior visual knowledge into deep models under low-contrast industrial environments, we adapt a bounding box-guided pseudo-mask generation method. Furthermore, an enhanced multi-scale defect feature optimization module (C2f_MSAA) is integrated to strengthen the feature extraction ability for multi-scale industrial defects, complemented by a dynamic collaborative spatial-channel reconstruction convolution (DC-SCConv) to facilitate dynamic feature reweighting. Experimental results on the SD-Saliency-900 and GC10-DET datasets evaluate the performance of the SCT-YOLO model, indicating its potential applicability to various industrial defect detection tasks. By transitioning from conventional deep architectures to a multi-dimensional prior feature-guided paradigm, our core research goal is to provide a more robust and precise vision-based solution for automated industrial production.

## 2. Related Work

### 2.1. Low-Level Features

In computer vision, image features are categorized into low-level and high-level features based on abstraction levels [11,12]. Low-level features are directly computed from pixel values with minimal semantic interpretation, describing local statistical or geometric properties of images, with shape, texture, and color being the most representative. Shape features describe the contours and geometric structures of targets and exhibit strong robustness to changes in illumination and color [13]; color features reflect the chromaticity and brightness distribution of pixels, are intuitive and computationally efficient, and have high sensitivity to specific targets [14]; texture features characterize the spatial arrangement patterns of surface microstructures and can effectively describe subtle surface changes [15,16]. In industrial surface defect detection, these low-level features directly correspond to physical defect manifestations, such as linear cracks, colored corrosion, and irregular pit textures.

High-level features, in contrast, are learned via multi-layer nonlinear transformations in deep networks [17]. Traditional detection methods prioritize high-level semantic features but often lose critical low-level details during propagation. Moreover, the learned feature distributions lack clear correlation with human-intelligible visual cues. For industrial defect detection, defect essence is primarily reflected in low-level attributes rather than high-level semantics. This paper innovatively re-examines the value of low-level features and enables the network to simultaneously take into account high-level semantic understanding and low-level detail perception by explicitly introducing low-level feature information such as shape, texture, and color into the detection process within the neural network.

### 2.2. Deep Learning-Based Industrial Surface Defect Detection Methods

Industrial surface defect detection is a core component of intelligent manufacturing quality control, requiring accurate and rapid defect identification in complex production environments [18]. Visual inspection has become the mainstream technology due to its cost-effectiveness and wide applicability, with successful applications including smartphone glass screen defect detection [19], while non-destructive testing methods (e.g., ultrasonic, radiographic testing) [20] are limited to offline high-precision scenarios due to high costs and low efficiency. Deep learning-based methods automatically learn discriminative features from raw images, eliminating the need for handcrafted features. Among computer vision tasks, object detection plays a core role in industrial defect detection as it provides both defect location and category information. Object detection algorithms are divided into two-stage and one-stage detectors: two-stage detectors (e.g., Faster R-CNN) [21] achieve high accuracy but low speed by first generating region proposals then performing classification and regression, while one-stage detectors directly regress target information, with the YOLO series dominating real-time industrial scenarios due to superior efficiency.

As shown in Table 1, YOLOv8 [22] is a classic lightweight model in the YOLO series. It adopts Anchor-Free design and decoupled detection head, balancing feature extraction efficiency and detection accuracy with a lightweight parameter scale, making it a basic improvement framework for downstream detection tasks. YOLOv10 [23] achieves NMS-free end-to-end training via consistent dual assignment strategy and optimizes decoupled head loss function, effectively reducing inference latency and breaking the accuracy-speed balance bottleneck. YOLO-World [24] innovatively introduces open-vocabulary detection, recognizing unseen categories via cross-modal feature alignment and breaking traditional models’ reliance on fixed training data, expanding application boundaries. More recently, YOLOv11 [25], released in late 2024, further streamlines the C2f module architecture and introduces a decoupled training strategy, achieving a significant improvement in small object detection accuracy while maintaining an equivalent parameter count. Released in 2025, YOLOv12 [26] adopts a novel attention-centric architecture design, and achieves further breakthroughs in both accuracy and speed via dynamic spatial attention mechanisms and cross-level feature fusion, establishing itself as the latest benchmark model in the field of industrial defect detection.

These deep learning-based surface defect detection methods offer efficient and accurate solutions for industrial quality inspection. They effectively solve complex scenarios and few-shot problems that traditional methods struggle with, and have significantly advanced the development of quality control technologies in intelligent manufacturing. The current technical challenges and industrial demands also provide clear improvement directions and practical value for the model architecture innovation and module optimization proposed in this paper.

### 2.3. Attention Mechanisms

Attention mechanisms, inspired by human visual selective attention, enable neural networks to adaptively focus on key input features and suppress irrelevant interference. In computer vision, they enhance model perception by dynamically reweighting feature responses, and are mainly divided into channel, spatial and self-attention based on focus dimensions. Single-dimensional attention often fails to balance local details and global context, so synergistic combinations are widely used. The C2f_MSAA and DC-SCConv proposed in this paper achieve precise perception of complex industrial defects via spatial-channel synergistic optimization while maintaining lightweight characteristics.

Each type optimizes features from distinct dimensions with classic applications: Channel attention emphasizes inter-channel importance differences. One classic example is SENet [27], which learns channel-wise weights via squeeze-and-excitation to amplify informative channels. The channel branch of CBAM [28], refines this strategy to improve feature selection accuracy. Spatial attention locates key target regions; the Spatial Attention Module [29] and CBAM’s spatial branch generate spatial weight maps to enhance target responses. Self-attention captures global pixel dependencies, with the Non-Local module [30] being a typical example that mines global semantic correlations.

### 2.4. Dual-Backbone Networks

Traditional object detection models mostly adopt single-backbone architectures, processing all visual information through a unified path with high computational efficiency and controllable parameter scales. Mainstream backbones include: Darknet (YOLO series backbone) balancing accuracy and speed; VGG [31] constructing basic representations via stacked 3×3 convolutions; ResNet [32] breaking depth limitations with residual connections; lightweight GhostNet [33] and ShuffleNet [34] optimizing efficiency via linear transformation and channel shuffling, respectively. However, single-backbone networks have limited capability in handling heterogeneous information and fusing multi-modal data, failing to fully exploit complementarity between different attributes.

In contrast, dual-backbone networks capture complementary visual features via parallel extraction paths, enhancing discriminative ability for complex patterns and obtaining richer joint representations than single backbones. This design supports customized feature extraction for different inputs but faces challenges of increased computational overhead and fusion mechanism design. In this paper, we construct the DSCA (Dual-Stream Collaborative Architecture) based on dual-backbone networks, which simultaneously utilizes original scene details and low-level prior feature maps, achieving information complementarity via the modulated fusion module and improving defect perception and discrimination capabilities.

## 3. Experimental Datasets

**Figure 1:**
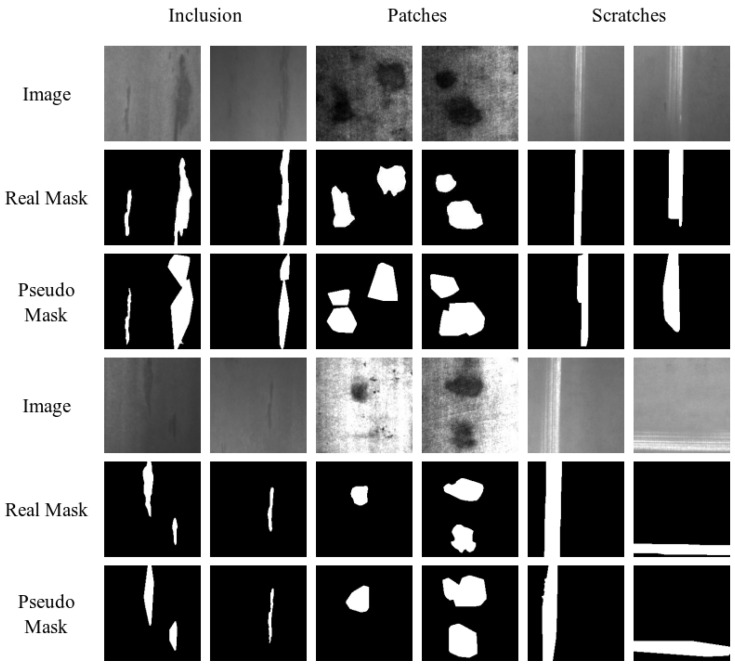
Comparison examples of the SD-Saliency-900 dataset, pseudo-masks and real masks.

The experiments in this paper were conducted on the SD-Saliency-900 [35], a benchmark dataset for steel surface defect detection. The original dataset was constructed and publicly released by the research team of Northeastern University and is widely used for evaluating steel surface defect detection methods. It originates from hot-rolled steel strip inspection scenarios and focuses on pixel-level annotation of salient defect regions. The SD-Saliency-900 dataset contains 900 steel surface defect images, covering three typical defect categories, namely Inclusion, Patches, and Scratches, with 300 samples for each category. Figure 1 presents representative samples and their corresponding ground-truth annotations from the SD-Saliency-900 dataset. For each data group, the first row shows the original defect image, and the second row displays the corresponding real mask.

The dual-stream collaborative defect detection model SCT-YOLO designed in this paper needs to incorporate the prior feature information of defect shape, texture, and color. Industrial defect detection scenarios impose stringent requirements on detection stability and accuracy, yet existing methods still struggle to achieve reliable defect identification under complex industrial conditions. The detailed process of this method is presented in Section 3.1.

### 3.1. Bounding Box-Guided Pseudo-Mask Generation

**Figure 2:**
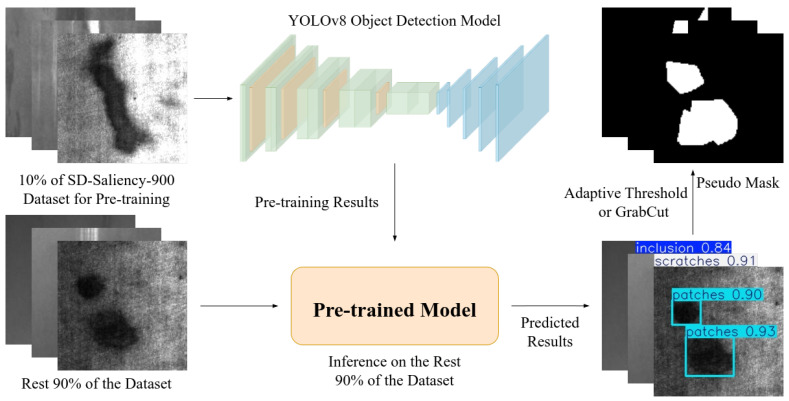
Pseudo-mask generation pipeline.

To provide defect region guidance for subsequent multi-dimensional feature extraction without relying on real masks, this study designs a pseudo-mask generation pipeline. This method does not participate in the training of the main model and only serves as a data preprocessing step. The workflow of this method is illustrated in Figure 2.

First, we strictly partition approximately 10 % of the samples from the original SD-Saliency-900 dataset for the pre-training of the YOLOv8 object detection model. These samples are completely isolated from all subsequent experimental data to ensure no risk of data leakage. Then, we use the pre-trained YOLOv8 model to perform object detection inference on the remaining 90 % of the data, generating coarse-grained defect prediction boxes as guidance information for pseudo-mask generation. It should be noted that the YOLOv8 model here is only used in this section and does not provide any assistance for subsequent experiments.

Next, we perform pixel-level segmentation on the regions within each prediction box. We automatically select the segmentation strategy according to the gray-level distribution characteristics of the regions inside the boxes: for regions with low contrast or complex textures, we first extract features from the prediction box as the initial input, then invoke the GrabCut algorithm [36] to perform iteratively refined segmentation. This algorithm automatically segments the foreground and background by minimizing an energy function based on the Markov Random Field, which is defined as follows:

(1) $$E\left(\alpha ,k,\theta ,Z\right)=\sum_{i}D\left(\alpha_{i},k_{i},\theta ,z_{i}\right)+\lambda \sum_{(i,j)\in N}S\left(\alpha_{i},\alpha_{j},z_{i},z_{j}\right)$$

Herein, α denotes the foreground/background label vector, D(·) represents the data term that measures the fitting degree between pixel colors and the Gaussian Mixture Model, S(·) is the smooth term that encourages adjacent pixels with similar colors to share the same label, and λ is the weight parameter balancing the two terms. Through the iterative optimization of this energy function, an accurate target segmentation mask can be obtained. By combining the adaptive thresholding method and the GrabCut algorithm, we can thus achieve the foreground region estimation within each prediction box.

Through observation of the dataset, we find that the three defect types, namely Inclusion, Patches, and Scratches, exhibit a block-distributed pattern. When using the adaptive thresholding method and GrabCut to obtain foreground region estimation, the defect regions may be segmented into multiple small fragmented regions. To address this issue, we need to merge the multiple independent foreground regions obtained in the previous step. We calculate the centroid distance and bounding box distance between every pair of regions; if the comprehensive distance is less than the set threshold, these regions are determined to belong to the same defect area and merged into a single connected region, thereby forming the final pseudo-mask for defect region segmentation. The schematic diagrams of the defect region segmentation pseudo-masks are presented in the last row of each sample group in Figure 1, labeled as Pseudo Mask.

It is worth noting that the segmentation fidelity of the generated pseudo-masks inevitably affects the quality of the extracted SCT features. If the pseudo-masks suffer from over- or under-segmentation, minor noise may be inherited by the color and texture descriptors. To mitigate this potential issue, the hyperparameters for pseudo-mask generation in this section were meticulously optimized through extensive grid searches to achieve a robust balance between segmentation reliability and execution efficiency. To quantitatively verify this effect, our internal oracle experiment indicates that while the baseline (YOLOv8n) yields 84.3% mAP, our framework achieves 88.9% mAP with pseudo-masks, which further increases to 90.2% mAP if flawless ground-truth (GT) masks are applied. Although utilizing human-annotated ground-truth masks could theoretically establish a performance upper bound, it relies on prohibitive manual labor costs and introduces severe data leakage risks during training, which contradicts the practical purpose. Therefore, our pseudo-mask strategy serves as a highly pragmatic and effective alternative for real-world applications.

As a key component of data preprocessing, this pseudo-mask generation scheme for detection and segmentation can automatically generate a large number of accurate pixel-level pseudo-labels with only a small amount of bounding box annotations, which significantly reduces the annotation cost in industrial scenarios and provides reliable support for defect region estimation in subsequent experiments.

### 3.2. Feature Image Generation Method

**Figure 3:**
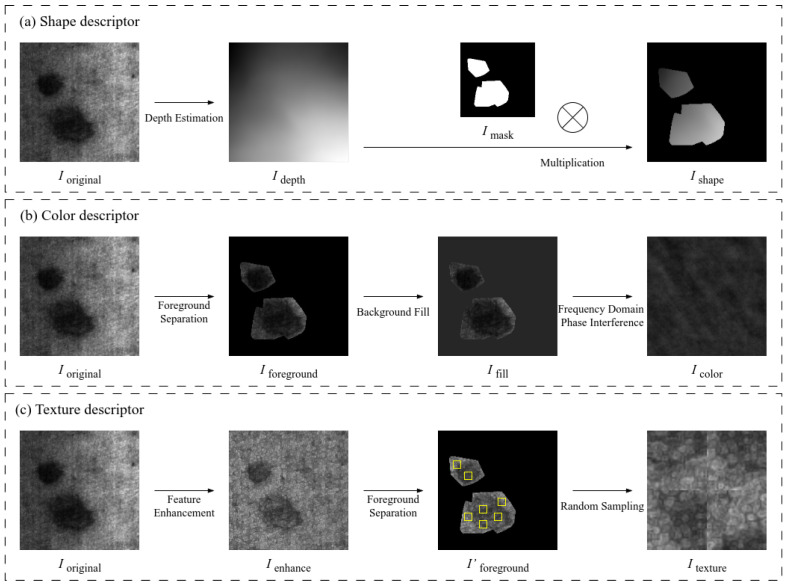
Process of extracting shape, color, and texture features.

Inspired by the work in [37], we investigate the shape, texture, and color features in defect detection. This section elaborates on the process of extracting these important defect features. The extraction pipeline of the three types of features is illustrated in Figure 3. The core difference between this work and previous studies lies in the fact that all features are extracted based on the pseudo-masks generated in Section 3.1 rather than real masks, so as to avoid the problem of data leakage.

For shape feature extraction, we first use the monocular depth estimation model DPT-Hybrid [38] to obtain the depth distribution of images. This model enables perception of the 3D geometric structure of defects and generates depth maps containing key 3D attributes such as surface undulations and depression depths, as illustrated in Figure 3a. Pixel-wise multiplication is performed between the depth map and the pseudo-mask to suppress the pixels in background regions, retaining only the depth information and 2D contours of defect regions [39], and finally generating a feature map focusing on defect shapes.

For color feature extraction, we first segregate the foreground defect regions from the original image using the pseudo-mask, calculate the average RGB values of these regions and fill them into the background regions to generate an intermediate image with a uniform background. To retain only the pure color features, we perform frequency-domain transformation on the filled image [40], randomly perturb the phase information in the frequency domain, and then convert it back to the spatial domain, as shown in Figure 3b. This process effectively destroys the spatial structure of the image while preserving the color statistical characteristics of the defect regions [41].

For texture feature extraction, a two-stage strategy is adopted. First, grayscale conversion, illumination normalization and multi-scale texture enhancement are performed on the original image, including small-scale sharpening, medium-scale smoothing and edge gradient enhancement. Then, four sub-regions of the same size are randomly sampled within the foreground regions defined by the pseudo-mask and stitched into a 2×2 composite image. To eliminate the interference from pseudo-features at the stitching boundaries, transition regions are set along the boundaries and weighted smoothing is applied [42], as shown in Figure 3c. This design preserves the local texture patterns while destroying the global shape information, making the extracted features focus on surface texture characteristics.

**Figure 4:**
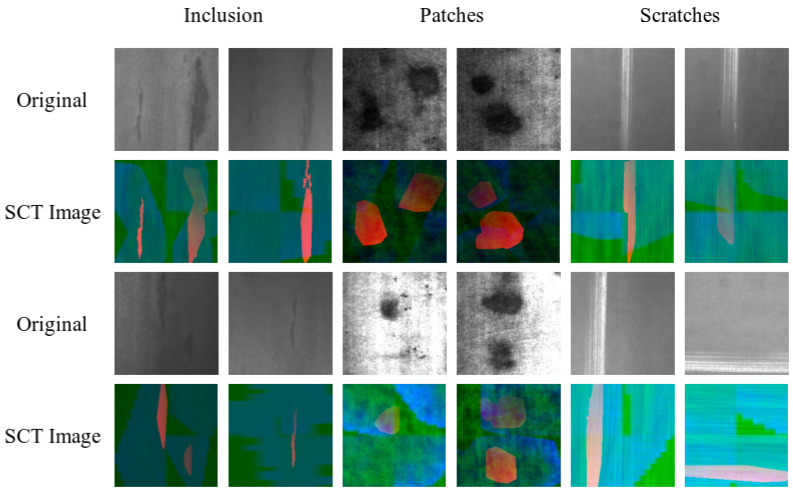
Comparison examples of original images and multi-Dimensional feature images. The distinct colors in the SCT images represent different channels mapped from the pseudo-color RGB space.

Through the above pipeline, the original image is converted into three types of complementary feature representations. To integrate these complementary cues into a unified input format for the subsequent dual-backbone network, the three independent feature maps are concatenated as individual RGB channels, yields a composite image rich in multi-dimensional defect characteristics. This process not only preserves the unique information of each feature but also facilitates the fusion of different features, which helps to enhance the model’s ability to understand and identify steel surface defects. Comparison examples of the original images and the Shape–Color–Texture (SCT) composite feature images are shown in Figure 4.

## 4. Proposed Method

### 4.1. Baseline Model: YOLOv8

Released by Ultralytics in 2023, YOLOv8 is an object detection framework that systematically integrates the advantages of previous-generation architectures and modular innovative designs, attracting sustained attention from both academia and industry. Its multi-level feature fusion mechanism and structural optimizations have achieved state-of-the-art performance in benchmark tests of three cross-modal tasks, namely image classification, object detection, and instance segmentation, which verifies the framework’s generalization capability in complex scenarios. The model integrates a backbone network based on the CSPDarknet structure, a feature enhancement network, and a detection head, which are responsible for feature extraction, multi-scale fusion, and bounding box prediction, respectively. By introducing a novel backbone network, an Anchor-Free detection head, and an optimized loss function, the model significantly improves deployment adaptability across hardware platforms in terms of inference speed and memory usage efficiency.

### 4.2. Improved YOLOv8 Object Detection Algorithm

**Figure 5:**
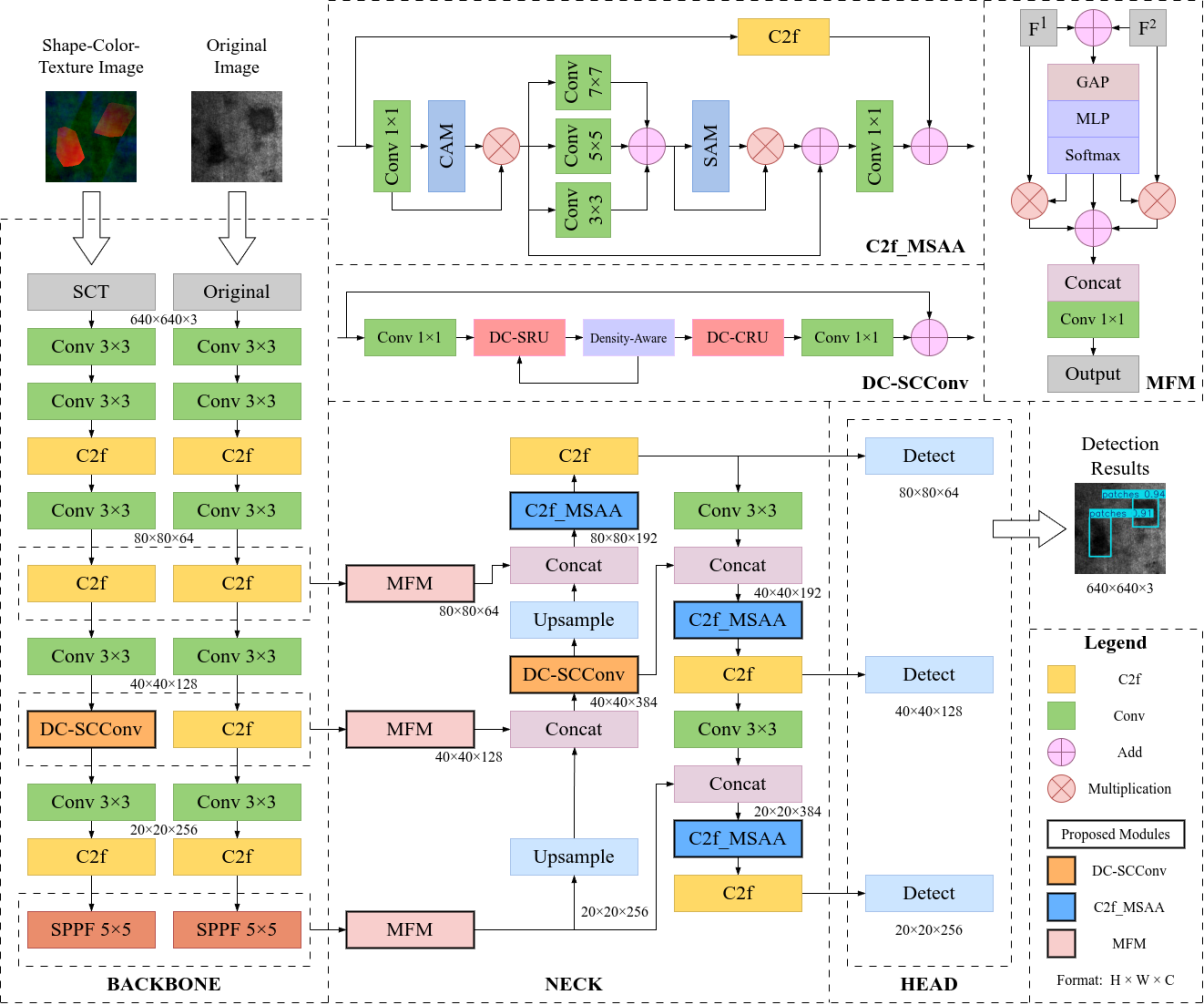
Architecture of SCT-YOLO network.

To effectively inject prior feature knowledge into the detection process, Industrial defect detection scenarios impose stringent requirements on detection stability and accuracy, yet existing methods still struggle to achieve reliable defect identification under complex industrial conditions. The model adopts a dual-backbone architecture, which processes the original images and the SCT multi-dimensional feature images (shape–color–texture multi-dimensional feature images) generated in Section 3.2, respectively. Figure 5 illustrates the detailed architecture of the proposed model. The two backbones extract features in parallel and enable multi-scale cross-backbone information interaction via the modulated fusion module (MFM). The merged features are finally fed into the detection head after subsequent operations to complete the defect detection task. This design enables the model to synergistically utilize raw visual information and prior feature knowledge, thereby improving detection performance.

### 4.3. Dual-Stream Collaborative Detection Architecture

Based on the YOLOv8n object detection framework, this study innovatively proposes the DSCA, consisting of two complementary backbone branches and a dynamic modulation-based fusion mechanism. The core design philosophy is to extract raw visual information and enhanced defect features through dual paths, and achieve adaptive feature fusion via MFM [43], thereby realizing information complementarity in industrial steel defect detection [32,44].

Specifically, the system adopts a dual-path parallel input structure: one path takes in raw steel surface defect image data, and the other path takes in the SCT multi-dimensional feature images generated in Section 3.2. The two input channels are connected to independent backbone networks, respectively, forming a complementary feature representation system through differentiated feature extraction strategies.

In the backbone for raw images, the network follows the original YOLOv8n backbone architecture and progressively extracts spatial and semantic representations through multi-stage downsampling operations. The primary objective of this branch is to preserve the low-level visual details and high-level contextual semantic information of raw images, thereby maintaining robustness under complex industrial backgrounds. During feature extraction, the backbone performs progressive multi-stage feature learning, while CSP-based feature aggregation is adopted to ensure efficient feature propagation and stable optimization.

The SCT feature branch focuses on learning discriminative multi-dimensional defect representations. This branch also adopts the CSPDarknet structure and introduces the proposed DC-SCConv module at key stages. By jointly modeling spatial and channel dependencies, the branch enhances the representation capability of subtle defect characteristics and improves sensitivity to weak or low-saliency defects.

Although the two backbone branches adopt similar overall architectures, their parameters are independently optimized during end-to-end training without parameter sharing between branches. The entire dual-stream framework is jointly supervised by a unified detection loss, enabling the two branches to collaboratively learn complementary defect representations through the subsequent MFM-based adaptive fusion process.

To achieve robust fusion of multi-scale defect features in steel surface images, this paper employs an attention mechanism-based modulated fusion module at the end of the dual-backbone network. Although YOLOv8 adopts a PANet-based multi-scale feature fusion architecture, its feature propagation paths and fusion operations remain essentially static. Traditional FPN/PANet-based fusion strategies mainly adopt fixed feature propagation paths and static feature aggregation mechanisms. Feature fusion is typically implemented through direct concatenation operations, where the contributions of different feature branches are implicitly treated equally. Such static fusion strategies cannot dynamically adjust the importance of heterogeneous semantic and explicit defect representations according to the discriminability of defect features.

In industrial visual inspection scenarios, defects such as scratches, holes, and rust spots on steel surfaces often exhibit significant variations in morphology, scale, and contrast against complex backgrounds. As a result, static fusion strategies may introduce redundant background texture information or weaken subtle defect responses, thereby limiting the representation capability of the network for challenging defect patterns.To address these limitations, the MFM introduces a dynamic weight generation mechanism to adaptively modulate and fuse features from the raw-image semantic branch and the SCT explicit feature branch. During the fusion process, the module autonomously strengthens salient defect features while suppressing irrelevant background texture information of the steel substrate, thereby improving the discriminability and robustness of feature representations.

**Figure 6:**
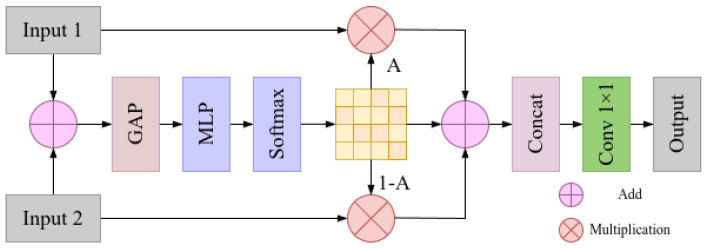
Structure diagram of the MFM.

The structure of the MFM is illustrated in Figure 6. Its core design philosophy is to generate spatial and channel-adaptive weights for the features to be fused via a dedicated attention sub-network, thereby achieving effective guided weighted fusion and improving overall feature representation quality.The module takes as input the feature maps from the two backbone networks, denoted as $F^{1}\in R^{B\times C\times H\times W}$ and $F^{2}\in R^{B\times C\times H\times W}$, respectively. First, the module feeds them into convolutional layers for preliminary adjustment, and then concatenates them along the channel dimension to form aggregated features. To achieve dynamic modulation, the core of the module is a parallel sub-network. This sub-network performs global average pooling on the concatenated aggregated features to capture the global contextual information of the feature maps and compresses it into a feature vector. This vector is then subjected to nonlinear transformation via a multi-layer perceptron composed of fully connected layers, so as to model the complex dependencies between feature channels and spatial locations. The transformed features are normalized using the Softmax function, finally generating a spatial-channel attention weight matrix $A\in R^{B\times height\times C\times H\times W}$, where height=2 represents the number of feature branches, and the spatial dimensions are H=1 and W=1. This weight matrix quantifies the relative importance of different feature branches and channels for the current defect discrimination task.Finally, the module modulates and fuses the two original input features using the generated dynamic weight matrix. The weight matrix *A* is multiplied element-wise by the feature $F^{1}$ to enhance the feature responses of key regions; meanwhile, its complementary weight 1−A is multiplied by the feature $F^{2}$ to fuse complementary information. The results of the two multiplications are summed to obtain the fused features, which are further integrated through a convolutional layer, ultimately outputting a feature map with enhanced discriminability. The entire process can be formally expressed as follows:

(2) $$F_{\mathrm{out}}=\mathrm{Conv}\left(A⊗F^{1}+\left(1-A\right)⊗F^{2}\right)$$

Herein, ⊗ denotes element-wise multiplication, and Conv represents the final integrating convolution.

The innovativeness of this architecture is mainly reflected in two aspects. First, the dual-path input design breaks through the limitations of traditional single-backbone networks by jointly modeling raw semantic information and explicit SCT defect representations. Through the collaborative learning of implicit visual semantics and explicit multi-dimensional defect cues, the proposed architecture enhances the completeness and discriminability of feature representations for steel surface defects.

Second, the MFM enables adaptive fusion of heterogeneous features from the two branches. During training, the module dynamically adjusts feature responses according to the discriminability of different defect patterns. For low-saliency or weak-contrast defects, the SCT feature branch tends to provide more discriminative complementary cues, while the raw image branch preserves richer contextual information in complex industrial backgrounds. This adaptive modulation mechanism improves the robustness and accuracy of defect representation learning.

### 4.4. Cross-Stage Multi-Scale Attention Aggregation Module

**Figure 7:**
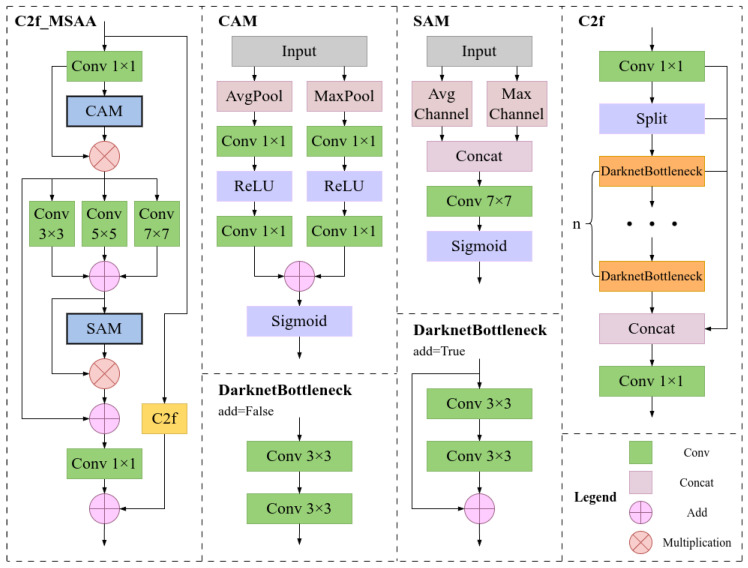
Structure diagram of C2f_MSAA.

To address the problems of huge scale differences in steel surface defects and complex background interference in industrial scenarios, we propose C2f_MSAA (Cross-stage Multi-Scale Attention Aggregation Module). By dynamically integrating channel and spatial attention mechanisms, this module [45] achieves adaptive enhancement of defect features and cross-stage collaborative optimization, significantly improving the model’s perception capability and detection accuracy for micro defects. The structure diagram of the module is illustrated in Figure 7.

The module has two parallel feature processing paths. In the first path, the input features are first compressed to the target dimension through a downsampling layer to focus on key channel information. Then, the CAM performs channel-level weighting on the feature maps, generates complementary channel importance maps through average pooling and max pooling, and produces channel attention weights after processing via a shared convolutional network and Sigmoid activation. This weight is multiplied element-wise by the original features to realize calibration of different feature channels.

The features enhanced by channel attention then enter the multi-scale convolution branch, which extracts local features of different receptive fields in parallel to capture local micro features, medium-scale features, and global contextual information of steel surface defects, respectively. This multi-scale design enables the module to perceive defect morphologies of various sizes simultaneously. The results of three-path feature extraction are initially fused through feature addition to form multi-scale aggregated features.

To further optimize the spatial distribution of features, the fused features are fed into the SAM. This mechanism calculates the mean and maximum values of the feature maps along the channel dimension, respectively, generating two single-channel feature maps that reflect the response intensity of features at different spatial positions. These two feature maps are concatenated along the channel dimension and then fused with contextual information through a 7×7 convolutional layer, finally generating spatial attention weights after Sigmoid activation. This weight is multiplied element-wise by the multi-scale aggregated features to achieve spatial focusing on potential defect regions. Finally, the aggregated features are adjusted for the number of channels through a convolutional layer to output the enhanced features.

The second path is constructed based on the C2f structure to realize cross-stage feature fusion. This path first adjusts the feature dimension through a 1×1 convolution, then uses the Split operation to divide the features into multiple branches, among which some branches are processed through several DarknetBottleneck [46] modules. The processed features of each branch are concatenated along the channel dimension and then reorganized through a 1×1 convolution to form cross-stage fused features.

In summary, C2f_MSAA is not a simple combination of existing components, but a customized architecture specifically optimized to overcome the limitations of standard attention mechanisms in industrial deployment. A standard Convolutional Block Attention Module (CBAM) processes features in a rigid, direct sequence (Channel-to-Spatial), which tends to over-amplify background clutter and noise when applied straight to raw steel surface images. To address this, C2f_MSAA rearranges the feature topology within the first parallel path: the CAM first filters channel-level background noise, the subsequent multi-scale branch explicitly extracts geometric cues across different receptive fields to capture varying defect sizes, and finally, the SAM locks onto the precise spatial boundaries. Concurrently, the second path retains the baseline C2f branch to preserve continuous cross-stage gradient flow. This hybrid parallel design allows the network to effectively isolate fine-grained industrial defects without introducing excessive computational overhead.

### 4.5. Dynamic Collaborative Spatial-Channel Reconstruction Convolution

SCConv is an efficiently compressed convolutional module characterized by its ability to suppress spatial and channel redundancy of feature maps. It significantly reduces the model parameter scale and computational cost while maintaining or even improving the feature learning capability of the original model [47]. However, the original SCConv adopts fixed thresholds, which makes it difficult to adapt to the dynamic distribution characteristics of different steel surface defects. To address this issue, this study proposes the DC-SCConv (Dynamic Collaborative Spatial and Channel Reweighting Convolution) module. By constructing an adaptive parameterized learning framework based on feature semantic density, it achieves dynamic collaborative optimization of weight allocation in both spatial and channel dimensions.

**Figure 8:**
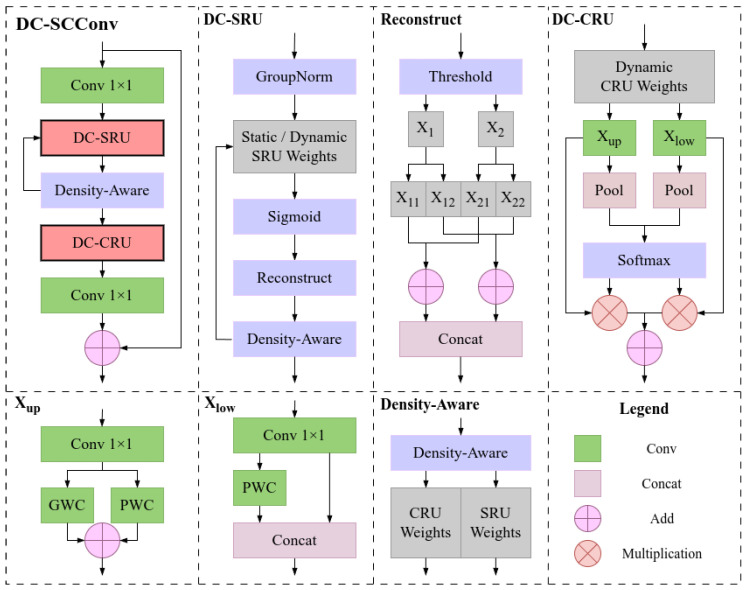
Structure diagram of DC-SCConv.

The core of DC-SCConv lies in a density-aware feedback control mechanism [48,49]. First, the local entropy of the feature map is calculated to quantify the regional feature complexity; a higher entropy value indicates that the feature distribution in this region is discrete and the semantic information is dense. Based on this, the entropy signal is converted into dynamic modulation coefficients through parameterized nonlinear mapping, which autonomously optimizes the weight allocation strategy of spatial and channel attention. According to the gradient change in local entropy values, high-entropy regions enhance the representation capability of high-frequency details in the spatial dimension, while low-entropy regions prioritize strengthening cross-channel semantic aggregation in the channel dimension. This forms a closed-loop feedback control system to improve the flexibility of feature selection. The block diagram of this module is shown in Figure 8.

For the spatial reconstruction unit, a separation-reconstruction operation is adopted. Separation aims to separate feature maps with rich information from those with scarce spatial information. Group Normalization [50] is used to group channels before normalization calculation. Values within each channel group share the same mean and variance, and the formula is as follows:

(3) $$\mathrm{GN}\left(x\right)=\frac{x-\mu_{G}}{\sqrt{\sigma_{G}^{2}+\epsilon }}\cdot \gamma +\beta$$

In the formula, *G* denotes the number of groups. $\mu_{G}$ and $\sigma_{G}^{2}$ represent the mean and variance of each channel group, respectively. γ and β are learnable scaling and shifting parameters.

Subsequently, we separate the reweighted feature maps using a Sigmoid gating unit. Weights above the threshold are set to 1 to obtain information-rich weights $W_{1}$, while weights below the threshold are set to 0 to obtain information-poor weights $W_{2}$. To further reduce spatial redundancy, a reconstruction operation is performed on the separated feature maps. The separated feature maps are then reconstructed: the two types of features are summed up, and information flow is enhanced through cross-reconstruction operations. The reconstructed feature maps are input into the density-aware feedback module.

The mathematical model of the density-aware feedback regulation mechanism can be expressed as follows. Given an input feature map $F\in R^{C\times H\times W}$, its local entropy matrix $H\in R^{C\times H\times W}$ is first calculated to quantify the regional feature complexity:

(4) $$H\left(x,y\right)=-\sum_{i=1}^{K}p_{i}\left(x,y\right)\log\left(p_{i}\left(x,y\right)\right)$$

Herein, $p_{i}\left(x,y\right)$ denotes the normalized distribution probability of feature values within the K×K neighborhood centered at coordinate (x,y). In the scenario of steel defect detection, the defect edge regions exhibit high entropy values due to their discrete feature distribution and sharp gradient changes. In contrast, feature-homogeneous background regions have gentle gradients and thus low entropy values.

Subsequently, the entropy signal is converted into dynamic adjustment factors α and β via the differentiable mapping function G(·):

(5) $$\left[\alpha ,\beta \right]=G\left(H;\theta \right)=\sigma \left(W_{2}\cdot \mathrm{ReLU}\left(W_{1}\cdot \mathrm{AvgPool}\left(H\right)\right)\right)$$

Herein, $\theta =\{W_{1},W_{2}\}$ denotes the learnable parameter matrices, and σ(·) represents the Sigmoid activation function. These dynamic factors adaptively adjust the attention weight allocation strategy for spatial and channel dimensions based on the semantic density distribution of the feature maps. α and β denote the spatial and channel dynamic adjustment factors, respectively. When the local entropy value is high, the adjustment factor α automatically increases to enhance the detail modeling capability in the spatial dimension; conversely, β is used to strengthen semantic integration in the channel dimension. The generated parameters are fed back negatively to the spatial reconstruction unit to adjust the threshold of the gating unit, enabling parameter adaptability without a significant increase in computational complexity.

The parameters calculated by the density-aware module are input to the channel reconstruction unit, where channel reconstruction of the feature maps is performed. $X_{\mathrm{up}}$ denotes the upper-layer transformation map, which is processed by grouped weight-wise convolution (GWC) and point-wise convolution (PWC), respectively, to extract rich high-level feature information. $X_{\mathrm{low}}$ denotes the lower-layer transformation map, which processes the remaining feature maps via PWC to extract complementary detail information. GWC performs independent convolution on grouped channels to reduce computational complexity; PWC achieves cross-channel information fusion to compensate for the information loss caused by channel grouping [51]. This design enables DC-SCConv to dynamically adjust its processing strategy based on the semantic density of input features, providing the system with feature extraction capability that is more adaptive to the characteristics of steel defects.

## 5. Experimental Results and Analysis

This section verifies the effectiveness and generalization ability of the improved network through systematic experiments. First, multiple ablation experiments are designed to decompose and verify each improved module, quantitatively analyzing the impact of each module on model performance. To further validate the algorithm’s advantages, the proposed SCT-YOLO is compared with mainstream object detection models such as YOLOv10 and Faster R-CNN, as well as other YOLOv8 improved models, across multiple metrics. The core evaluation metrics include mAP@0.5 (mean Average Precision), Params (number of parameters), FPS (frames per second), and GFLOPs (giga floating-point operations per second), which comprehensively assess the model performance in defect detection tasks. In addition, combined with the visualization of detection results, the improvement effect of the algorithm relative to the baseline model is intuitively demonstrated. Finally, the generalization ability of the improved network on diverse datasets is verified on a new steel defect dataset GC10-DET.

### 5.1. Experimental Configuration and Evaluation Metrics

All experiments in this study are conducted in a unified hardware and software environment to eliminate systematic biases. The experimental platform is configured with an Intel Core i7-13700K processor, an NVIDIA GeForce RTX 4080 graphics card with 16 GB video memory, and 64 GB RAM, running on the Ubuntu 20.04.6 LTS operating system. The algorithm is implemented based on the PyTorch 2.0.0 deep learning framework. The model training adopts the SGD (Stochastic Gradient Descent) optimizer with an initial learning rate of 0.01 and a batch size of 16, for a total of 500 epochs. All comparative experiments are performed under the same hyperparameter configuration to ensure fair evaluation of model performance and the reliability of experimental results. Regarding the classification loss, the label smoothing factor was maintained at 0.0 (i.e., disabled) in accordance with the standard default configurations of the vanilla YOLOv8 framework, as no severe overfitting was observed during the initial baseline training phase.

Experiments are carried out on the SD-Saliency-900 steel defect dataset introduced earlier. After excluding the 10% of data used in Section 3.1, the remaining 90% of data is divided into training and validation sets at an 8:2 ratio using stratified random sampling to meet the requirements of model training and validation. Each defect category contains 216 samples in the training subset and 54 samples in the validation subset. All experimental data are processed using the shape-texture-color defect feature extraction pipeline proposed in Section 3, ensuring that the input features match the dual-backbone input requirements of the SCT-YOLO model.

This paper comprehensively evaluates model performance using multi-dimensional metrics. For detection accuracy, we adopt mAP@0.5 and mAP@0.5–0.95 as evaluation metrics, with mAP@0.5 designated as the core metric that comprehensively reflects the model’s localization and classification capabilities at an Intersection-over-Union (IoU) threshold of 0.5; mAP@0.5–0.95 represents the average mAP across IoU thresholds ranging from 0.5 to 0.95 with a step of 0.05. Model efficiency and complexity are measured by the number of parameters, computational complexity, and inference speed. Among them, Params refers to the total number of trainable parameters of the model, which is related to the storage overhead and deployment cost; GFLOPs, expressed in billions of floating-point operations, is used to evaluate the theoretical computational complexity of forward propagation; the inference speed is represented by FPS, measured in the above experimental environment, to evaluate the real-time processing capability of the model. In addition, the power consumption during inference is further evaluated to reflect the energy efficiency of different models. The average GPU power of NVIDIA RTX 4080 Super is estimated based on runtime utilization, ranging from 180 W to 290 W depending on model complexity and GPU occupancy. The energy consumption for processing 1000 images is calculated as:

(6) $$E=\frac{P_{\mathrm{avg}}\times 1000}{\mathrm{FPS}\times 3600}$$

where $P_{\mathrm{avg}}$ denotes the average GPU power, FPS is the inference speed, and 3600 converts seconds to hours. This metric enables a fair comparison of energy efficiency across different detection models under identical hardware conditions.

### 5.2. Effectiveness Analysis of the Proposed Modules

To verify the effectiveness of the proposed Dual Stream Collaborative Architecture and its core modules in the recognition of industrial steel surface defects, this study designs comparative experiments. Based on the same training strategy and hardware environment, the experiments quantitatively analyze the impact of each innovative module on model performance by gradually introducing them. The experimental results are shown in the tables below.

First, the effectiveness of the proposed DSCA architecture is verified in Table 2. When only the raw-image branch is used, the model achieves an mAP of 84.3%. After introducing the dual-backbone structure, the mAP further increases to 84.8%, indicating that the dual-stream parallel architecture can effectively utilize complementary visual information from different feature sources. However, the dual-backbone design also increases the number of parameters from 3.01 M to 4.39 M and the GFLOPs from 8.1 to 11.5, while the inference speed decreases correspondingly. Furthermore, after incorporating the SCT feature branch, the mAP improves to 85.4%, demonstrating that explicit multi-dimensional defect representations can significantly enhance the discriminability of defect features without introducing additional computational overhead.

To further improve the feature fusion capability between heterogeneous branches, the proposed MFM is introduced. Experimental results show that the MFM improves the mAP from 84.8% to 85.6% compared with the dual-backbone architecture without MFM, while the number of parameters increases by only 0.09 M. More importantly, when combined with the SCT features, adding MFM delivers a substantial synergistic improvement from 85.4% to 86.8% mAP (+1.4% mAP), achieving the peak overall accuracy. This sharp contrast demonstrates that MFM functions as a critical modulation bridge that adaptively aligns and scales low-level texture details and high-level semantic features across different feature spaces. Meanwhile, the GFLOPs remain nearly unchanged and the inference speed slightly increases from 374.6 FPS to 381.2 FPS. These results indicate that this adaptive modulation mechanism can achieve more effective feature fusion and unlock the full potential of heterogeneous features without introducing significant additional computational complexity.

Finally, when the complete DSCA architecture is deployed, the overall mAP reaches 86.8%, representing a 2.5% improvement over the baseline model. Although the dual-stream architecture introduces additional computational overhead, resulting in increased parameters and reduced inference speed compared with the baseline model, the proposed framework still maintains 379.4 FPS with only 11.4 GFLOPs, demonstrating a favorable balance between detection accuracy and computational efficiency for industrial steel defect detection tasks.

In the experiments shown in Table 3, C2f_MSAA exhibits superior engineering adaptability compared with the basic module MSAA. Although their mAP values are similar, C2f_MSAA significantly reduces computational redundancy via cross-stage feature fusion, cutting parameters by 40.9% and GFLOPs by 59.6% while increasing inference speed by 27.4%. When deployed independently, this module maintains a high frame rate of 552.3 FPS and achieves an mAP of 86.6% (a 2.3% gain over the baseline). This demonstrates that C2f_MSAA delivers substantial multi-scale detection performance with reduced computational overhead, proving its high cost-effectiveness for steel defect recognition.

The data in Table 4 demonstrates the improvement advantages of DC-SCConv over SCConv. With only a marginal increase in parameters compared to SCConv, DC-SCConv improves mAP by 0.5% and boosts inference speed by 45.1% simultaneously, verifying the superiority of the dynamic collaborative mechanism over static feature reconstruction. This module’s adaptive weight allocation enhances feature representation while avoiding extra computational overhead. When deployed independently, DC-SCConv exhibits unique advantages: despite a 0.7% reduction in parameters relative to the baseline, it achieves a 2.1% mAP gain while maintaining a high inference speed of 460.9 FPS. This performance is attributed to enhanced robustness against surface noise, where the dynamic mechanism effectively suppresses false detections from background interference. Notably, DC-SCConv’s GFLOPs are 3.7% lower than the baseline, indicating it utilizes computing resources more effectively to yield detection gains, which is crucial for identifying micro-defects like fine scratches.

Overall, each proposed module exhibits distinct performance gains: the dual-backbone structure DSCA provides basic advantages, the MFM optimizes the efficiency of feature fusion, C2f_MSAA enhances multi-scale feature extraction capability, and DC-SCConv strengthens spatial-channel feature representation. These improvements jointly promote the balance between accuracy and efficiency of the model, providing a feasible technical solution for real-time defect detection in complex industrial scenarios.

### 5.3. Analysis of Ablation Experiment Results

To quantitatively evaluate the contribution degree of each module of the dual-stream collaborative detection architecture and its core modules to the performance of SCT-YOLO in the steel surface defect detection task, we designed systematic ablation experiments on the SD-Saliency-900 dataset, and the experimental results are presented in Table 5. We analyze the contributions of each module to detection accuracy, computational efficiency, and model lightweighting step by step. The experimental results show that the proposed dual-stream collaborative framework and its derivative module combination significantly improve the detection capability of key defects while meeting the real-time performance requirements for industrial deployment, and verify the flexibility and scalability of the architecture design at the same time.

To further verify the reliability and statistical significance of the ablation results, all ablation experiments were independently repeated five times using different random seeds. The mean ± standard deviation of mAP is reported in Table 5. In addition, paired *t*-tests were conducted between the baseline and the proposed method, demonstrating statistically significant improvements (p<0.05).

To quantify the contribution of each proposed component, we first evaluate their standalone performances, the details of which have been discussed in Section 5.2. The integration of these modules yields progressive improvements. While the individual efficacy of DSCA, C2f_MSAA, and DC-SCConv has been thoroughly validated, their combined performance reveals further synergistic effects.

Module combination experiments further revealed the core supporting role of the dual-stream architecture. When DSCA was combined with C2f_MSAA, the model’s mAP reached 87.4%, but the number of parameters increased to 5.75 M and the inference speed dropped to 261.7 FPS, reflecting that the computational cost of multi-scale modeling and dual-stream collaboration needs further optimization. When DSCA was deployed in conjunction with DC-SCConv, the mAP increased to 87.1%, and the number of parameters (4.39 M) was lower than that of DSCA used alone, indicating that the dynamic collaborative mechanism can effectively alleviate the redundant computation problem of the multi-stream architecture. Its spatial-channel reconstruction strategy enhanced the feature separability between defect regions and the background, highlighting the scalability of the dual-stream framework. The combination of C2f_MSAA and DC-SCConv exhibited lightweight advantages: the mAP increased to 87.5%, the number of parameters was close to that of the baseline model at only 3.00 M, the FPS reached as high as 674.2, and the GFLOPs remained at 8.0, which proves that the synergy of the two modules in feature optimization can balance accuracy and efficiency.

The collaborative combination of C2f_MSAA and DC-SCConv, based on the dual-stream, architecture achieves a significant comprehensive detection performance breakthrough. The mAP@0.5 of the complete model increases to 88.9%, achieving a substantial 4.6% improvement compared with the baseline model in detection accuracy. In this design, the dual-stream structure provides solid architectural support for multi-scale and multi-dimensional feature fusion, enabling C2f_MSAA to capture and refine cross-layer semantic information more effectively for various defect types, while DC-SCConv further enhances the feature discrimination and localization accuracy of complex and tiny defect regions through dynamic feature reconstruction.

Based on a comprehensive analysis of the ablation experiment results, DSCA was confirmed to be the core driving force for improving the steel defect detection performance of SCT-YOLO. Through the interaction mechanism of heterogeneous feature streams, it provides a basic framework for the efficient collaboration of C2f_MSAA and DC-SCConv. Benefiting from the cascade optimization and mutual coordination between different core modules, the proposed model achieves a significant 4.6% mAP improvement over the baseline method. Experiments demonstrate that the dual-stream design effectively enables significant improvements in detection accuracy and unlocks greater performance potential through inter-module collaboration. This approach provides an innovative and reliable solution for high-precision industrial steel surface defect inspection tasks.

**Figure 9:**
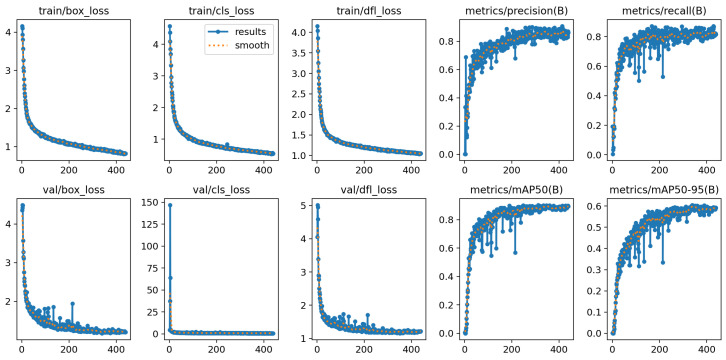
Loss-vs-epoch curves and convergence analysis of our model.

To further evaluate the training stability and convergence behavior of the proposed dual-stream network, the training and validation loss curves during the training process are presented in Figure 9.

As observed from the curves, both the training loss and validation loss decrease rapidly during the initial 100 epochs, demonstrating the efficient learning capability of our architecture. Throughout the subsequent training phase, the curves progress smoothly without violent fluctuations, and eventually reach a stable plateau. Notably, the validation loss consistently tracks the downward trend of the training loss and stabilizes synchronously, which firmly proves the absence of vanishing or exploding gradients and validates that our model is free from severe overfitting. This smooth and stable convergence verifies that the proposed dual-stream design possesses robust numerical stability during joint optimization.

### 5.4. Analysis of Comparative Experiment Results

**Figure 10:**
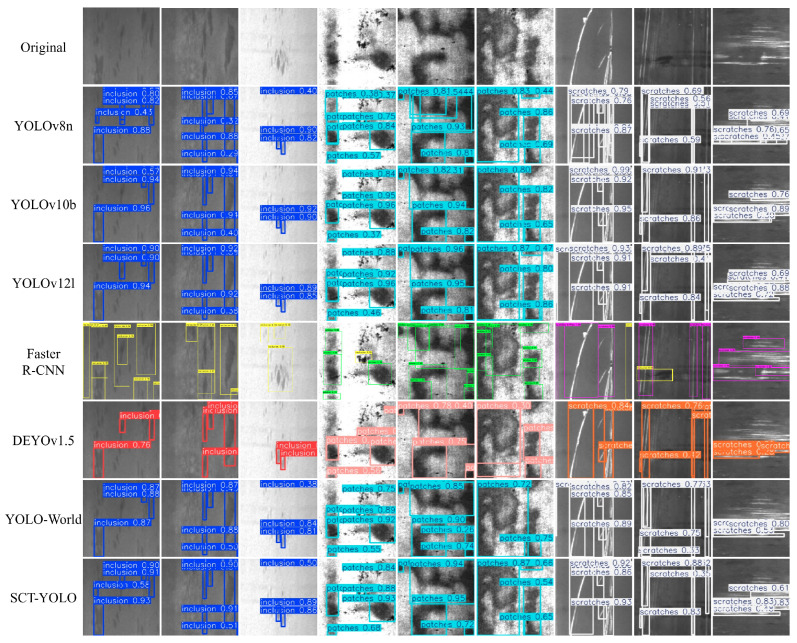
Visual comparison of detection results. The bounding boxes in various colors represent the detected defect regions and corresponding categories predicted by different models.

To comprehensively evaluate the overall detection performance of the SCT-YOLO model in the steel surface defect detection task, this study selects current mainstream detection models for horizontal comparison, as shown in Table 6. These models include one-stage detectors (partial models of the YOLO series), two-stage detectors (Faster R-CNN), and improved models (DEYOv1.5, YOLO-World). In addition, a further visual comparative analysis is presented in Figure 10. Existing models generally suffer from missed detection of small defects and false detection of background interference in complex industrial scenarios. In contrast, SCT-YOLO effectively alleviates such inherent detection drawbacks through its dual-stream collaborative architecture and dynamic feature reconstruction mechanism. The experimental results show that SCT-YOLO achieves excellent and robust high-precision detection performance in complex steel surface defect identification tasks, effectively reducing small-defect missed detection and background false detection and achieving significant performance improvements compared with other mainstream detection methods.

In terms of detection accuracy, SCT-YOLO significantly outperforms all comparative models with an mAP of 88.9%, representing a 4.6% improvement compared with the baseline model YOLOv8n. The visual comparison results presented in Figure 10 further demonstrate that existing models suffer from missed detection of defects with tiny morphologies or low contrast with the background, whereas SCT-YOLO exhibits more stable detection capability on such hard samples by virtue of the enhancement and focusing of prior features through its dual-stream collaborative architecture. In addition, existing methods improve accuracy by increasing model capacity (e.g., the parameter count of YOLOv10b reaches 20.41 M) or introducing complex attention mechanisms (e.g., the GFLOPs of YOLO-World reach 32.6). However, SCT-YOLO achieves accuracy breakthroughs with only 5.66 M parameters via the dual-stream collaborative architecture and dynamic reconstruction mechanism.

In the dimension of model lightweighting, SCT-YOLO demonstrates excellent parameter efficiency. Its parameter count of 5.66 M is 87.7% higher than that of the baseline model YOLOv8n, but the detection accuracy is significantly improved. In contrast, high-precision models such as DEYOv1.5-E (58.61 M) and Faster R-CNN (41.53 M) have parameter counts 10.4 times and 7.3 times that of SCT-YOLO, respectively, yet fail to achieve comparable detection accuracy. This comparison reveals the limitations of traditional models in improving performance through parameter expansion. In contrast, SCT-YOLO maximizes feature representation capability with limited parameters via the dual-backbone collaborative mechanism and dynamic feature reconstruction.

In terms of computational efficiency, SCT-YOLO maintains industrial-grade real-time detection capability with an inference speed of 257.2 FPS. Although this is lower than YOLOv8n’s 842.8 FPS, it is significantly superior to models at the same accuracy level. Compared with the two-stage detector Faster R-CNN (51.7 FPS), SCT-YOLO achieves a 397.49% speedup, reducing single-frame inference time from 19.3 ms to 3.89 ms. This performance fully satisfies the real-time detection requirements of industrial production lines. More crucially, the GFLOPs of SCT-YOLO is only 15.2, far lower than those of YOLO-World (32.6 GFLOPs) and YOLOv10b (98.0 GFLOPs), indicating that its computing resource utilization efficiency is significantly better than that of existing methods. The visual results in Figure 10 further confirm that while maintaining high inference speed, SCT-YOLO has stronger robustness to complex background interference. This proves that the strategy of existing methods to improve performance by stacking computing resources (e.g., YOLOv10b’s 98.0 GFLOPs) has significant efficiency bottlenecks, whereas SCT-YOLO achieves optimal allocation of computing resources through computing flow optimization and cross-stage feature reuse.

Regarding energy consumption, the heavy-weight detectors Faster R-CNN and DEYOv1.5-E exhibit relatively high overheads of 1.612 Wh and 1.036 Wh, respectively. In comparison, SCT-YOLO reduces the energy consumption to 0.216 Wh, which represents an 86.6% and 79.2% energy reduction compared to Faster R-CNN and DEYOv1.5-E, respectively. Although the ultra-lightweight YOLOv8n achieves the lowest energy consumption of 0.059 Wh, its detection accuracy is 4.6% lower in mAP than our model. These quantitative comparisons indicate that SCT-YOLO maintains a low energy overhead while achieving the highest detection accuracy, offering a well-balanced trade-off between computational cost and task performance.

Comprehensive comparative experiments and visual analysis show that SCT-YOLO effectively improves the overall detection effect and resolves the inherent detection difficulties in industrial defect detection via its dual-stream collaborative architecture. The model achieves a substantial and reliable improvement in core detection accuracy, and consistently delivers excellent identification results for various steel surface defects. Visual results further confirm that this method has more stable detection performance in complex background and tiny defect scenarios, with false detection and missed detection significantly reduced. These achievements reflect the prominent detection advantages and application value of the dual-stream collaborative architecture integrating prior features in industrial quality inspection tasks.

### 5.5. Generalization Performance Verification on the GC10-DET Dataset

GC10-DET [53] is a benchmark dataset for industrial steel surface defect detection, collected from real manufacturing inspection scenarios. It consists of gray-scale images captured from steel sheet surfaces under complex production conditions, including uneven illumination and strong surface reflections. The dataset contains ten typical types of steel surface defects, such as punching deformation, weld line defects, crescent-shaped gaps, water spots, oil spots, and scratches. According to the imaging characteristics and defect distribution, GC10-DET is more consistent with cold-rolled or galvanized steel sheet inspection scenarios, which typically require high-precision surface quality evaluation. After a rigorous process of data verification and quality cleaning on the originally collected samples, 2065 valid images were retained. These samples were then randomly partitioned into a training set (1858 samples) and a validation set (207 samples) at a ratio of 9:1.

It is worth noting that SD-Saliency-900 mainly originates from hot-rolled steel strip inspection scenarios, whereas GC10-DET is more consistent with cold-rolled or galvanized steel sheet inspection conditions. Since hot-rolled and cold-rolled steels exhibit significant differences in surface characteristics, defect morphology, and imaging conditions, evaluating the proposed method on both datasets helps verify its robustness and generalization capability across different industrial steel manufacturing processes.The experimental results are presented in Table 7. These results demonstrate that SCT-YOLO continues to demonstrate significant performance advantages on the GC10-DET dataset, achieving a 12.7% increase in mAP compared to the baseline YOLOv8n model.

In terms of detection accuracy, SCT-YOLO sets a new state-of-the-art performance on the GC10-DET dataset with an mAP of 78.4%, achieving a 2.0% mAP improvement over the second-best performing YOLO-World and a 9.1% gain over the lightweight improved model DEYOv1.5. Notably, the GC10-DET dataset suffers from local overexposure and shadow coverage artifacts caused by its multispectral imaging setup. These challenging conditions often lead to false detections in traditional models, such as mistaking reflective areas for crescent-shaped gaps. Experimental visualization results show that the original models have a certain false detection rate in high-reflection areas, while SCT-YOLO mitigates such false detections via its dynamic channel attention mechanism. This verifies the effectiveness of the cross-modal feature selection mechanism in the dual-stream collaborative architecture: by suppressing high-frequency noise responses in reflective regions, the model maintains stable detection accuracy even under complex illumination conditions.

In the dimension of model lightweighting, SCT-YOLO achieves an mAP of 78.4% with only 5.66 M parameters. Comparative experiments indicate that DEYOv1.5-E has a parameter count of 58.62 M, which is far higher than that of SCT-YOLO, yet its detection accuracy (69.3%) is significantly lower. This highlights the excellent control over parameter efficiency achieved by SCT-YOLO through its dynamic collaborative spatial-channel reconstruction convolution and dual-stream feature fusion mechanism while ensuring high-precision detection. For the newly added punching deformation defect detection task in the GC10-DET dataset, SCT-YOLO also achieves high detection accuracy, with an overall 12.7% improvement over the baseline model, confirming the strong generalization capability and engineering application value of this lightweight architecture in complex industrial defect detection scenarios.

In terms of computational efficiency, SCT-YOLO maintains real-time processing capability with 260.3 FPS on the GC10-DET dataset. Its inference speed is approximately 403.48% faster than that of the two-stage detector Faster R-CNN, and its GFLOPs are only 15.5% of those of YOLOv10b. In particular, targeting the high-dimensional characteristics of multispectral imaging data, SCT-YOLO implements intelligent compression of feature channels via the cross-stage multi-scale attention aggregation module. This proves that the architecture can effectively balance computing resource allocation and avoid the diminishing marginal returns problem of traditional models caused by blindly increasing computational complexity.

**Figure 11:**
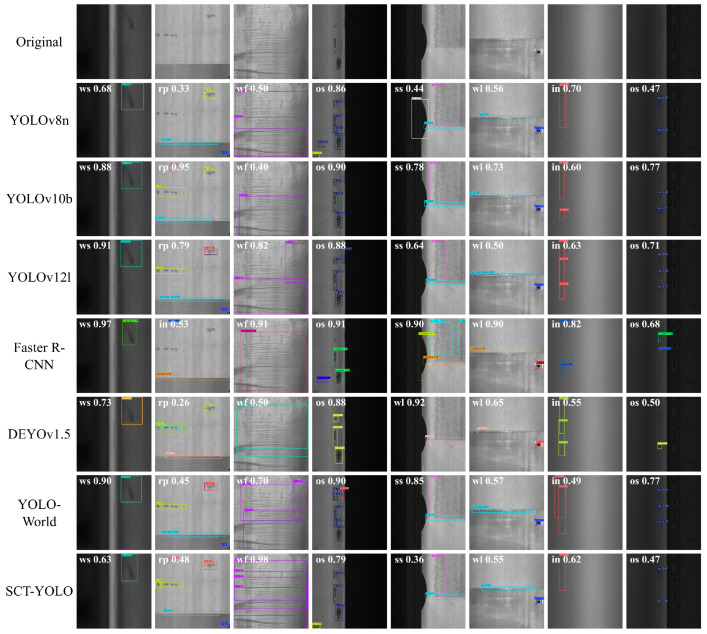
Visual Comparison of detection results on the GC10-DET Dataset. The bounding boxes in various colors represent the detected defect regions and corresponding categories predicted by different models. The abbreviated text and values in the upper-left corner of each sub-image provide an enlarged, clear display of the key detection results for better readability.

The visual comparison of detection results of different defect detection methods on the GC10-DET dataset is presented in Figure 11, which further reveals the engineering advantages of SCT-YOLO. For punching deformation samples covered by local shadows, YOLOv12l results in missed detections due to insufficient illumination adaptability, while SCT-YOLO reduces the missed detection rate through dual-stream feature extraction. In complex reflective scenarios, YOLO-World mistakenly identifies rolling textures as weld defects, whereas SCT-YOLO effectively reduces such false detections by virtue of its dynamic spatial-channel reconstruction mechanism. These results confirm that the cross-modal collaborative mechanism of SCT-YOLO can effectively overcome illumination interference in industrial imaging, and its detection robustness is significantly superior to that of existing methods.

Based on the above experimental results, it can be concluded that the excellent performance of SCT-YOLO on the GC10-DET dataset fully verifies the strong generalization capability of its architectural design, which achieves a competitive mAP accuracy of 78.4% for industrial defect detection tasks. Compared with traditional schemes relying only on simple data augmentation strategies, this model effectively improves the adaptability of industrial defect detection systems to complex imaging environments through dual-stream feature collaboration and dynamic feature reconstruction mechanisms.

## 6. Conclusions

This study introduces a dual-stream collaborative SCT-YOLO model that effectively integrates three-dimensional prior features of defect shape, color, and texture, thereby enhancing the accuracy of industrial surface defect detection. Distinct from traditional single-backbone defect detection methodologies, the primary scientific focus of this study is the formalization of a dual-stream collaborative architecture. This framework provides a potential perspective for defect representation by incorporating a cross-backbone multi-scale fusion mechanism to couple multi-modal physical priors with deep learned features. Experimental results demonstrate that the multi-dimensional feature-guided learning and cross-backbone multi-scale fusion significantly strengthen the representation of key defect characteristics, achieving robust detection performance. The model’s strong generalization across different steel surface defect datasets indicates its high potential for practical deployment. In subsequent research endeavors, our objective is to delve deeper into evaluating the applicability, adaptability, and scalability of this methodology across a broader spectrum of distinct industrial sectors (such as PCB components, textiles, or solar panels) to comprehensively validate its universal industrial applicability.

## Figures and Tables

**Table 1:** YOLO Series Versions and Key Innovations.

Version	Year	Key Innovation
YOLO	2016	Original real-time detection framework
YOLOv2	2017	Batch norm, anchor boxes, higher resolution
YOLOv3	2018	Multi-scale features, residual connections
YOLOv4	2020	Enhanced structure, better speed-accuracy trade-off
YOLOv5	2020	Lightweight CSP backbone, streamlined design
YOLOv8	2023	Anchor-free, decoupled head, simplified C2f
YOLOv10	2024	NMS-free end-to-end training, dual assignment
YOLO-World	2024	Open-vocabulary, cross-modal feature alignment
YOLOv11	2024	Optimized C2f, small-object enhancement
YOLOv12	2025	Attention-centric, dynamic spatial attention

**Table 2:** Comparative experimental results of DSCA and Baseline model. The “✓” indicates that the corresponding component is adopted. The “↑” and “↓” symbols represent performance improvement and decrement relative to the baseline, respectively.

Dual-Backbone	SCT Dataset	MFM	mAP/%	Params/M	FPS	GFLOPs
			84.3	3.01	842.8	8.10
✓			84.8 ↑0.5	4.39 ↑1.38	374.6 ↓468.2	11.5 ↑3.4
✓	✓		85.4 ↑1.1	4.39 ↑1.38	371.1 ↓471.7	11.5 ↑3.4
✓		✓	85.6 ↑1.3	4.48 ↑1.47	381.2 ↓461.6	11.4 ↑3.3
✓	✓	✓	86.8 ↑2.5	4.48 ↑1.47	379.4 ↓463.4	11.4 ↑3.3

**Table 3:** Comparative experimental results of C2f_MSAA and MSAA. The “↑” and “↓” symbols represent performance improvement and decrement relative to the baseline, respectively.

	mAP/%	Params/M	FPS	GFLOPs
Baseline	84.3	3.01	842.8	8.1
MSAA	86.4 ↑2.1	5.96 ↑2.95	433.5 ↓409.3	22.3 ↑14.2
C2f_MSAA	86.6 ↑2.3	3.52 ↑0.51	552.3 ↓290.5	9.0 ↑0.9

**Table 4:** Comparative experimental results of DC-SCConv and SCConv. The “↑” and “↓” symbols represent performance improvement and decrement relative to the baseline, respectively.

	mAP/%	Params/M	FPS	GFLOPs
Baseline	84.3	3.01	842.8	8.1
SCConv	85.9 ↑1.6	2.81 ↓0.20	317.7 ↓525.1	7.5 ↓0.6
DC-SCConv	86.4 ↑2.1	2.99 ↓0.02	460.9 ↓381.9	7.8 ↓0.3

**Table 5:** Ablation Experiment Results. The “✓” indicates that the corresponding component is adopted.

DSCA	C2f_MSAA	DC-SCConv	mAP/%	Params/M	FPS	GFLOPs
			84.3 ± 0.1	3.01	842.8	8.1
✓			86.8 ± 0.1	4.48	379.4	11.4
	✓		86.6 ± 0.1	3.52	552.3	9
		✓	86.4 ± 0.1	2.99	460.9	7.8
✓	✓		87.4 ± 0.1	5.75	261.7	15.4
✓		✓	87.1 ± 0.2	4.39	266.4	11.1
	✓	✓	87.5 ± 0.1	3.00	674.2	8
✓	✓	✓	88.9 ± 0.1 *	5.66	257.2	15.2

Note: Values are reported as mean ± standard deviation across five independent training runs. * denotes p<0.05 compared with the baseline model using a paired *t*-test, indicating statistical significance.

**Table 6:** Comparative experiment results.

	AP/%			mAP/%	Params/M	FPS	GFLOPs	Energy/Wh
	In	Pa	Sc					
YOLOv8n	87.6	85.2	80.2	84.3	3.01	842.8	8.1	0.059
YOLOv10b	85.0	85.3	87.5	85.9	20.41	197.7	98	0.337
YOLOv12l	86.0	85.0	87.2	86.1	26.34	143.9	88.6	0.502
Faster R-CNN	75.7	89.5	95.0	86.7	41.53	51.7	177.6	1.612
DEYOv1.5-E [52]	87.7	87.4	85.2	86.8	58.61	75.1	173.8	1.036
YOLO-World	88.1	85.9	87.8	87.3	12.75	377.1	32.6	0.169
SCT-YOLO	89.5	90.6	86.7	88.9	5.66	257.2	15.2	0.216

**Table 7:** Experimental results on the GC10-DET dataset.

	AP/%										mAP/%	Params/M	FPS	GFLOPs
	ph	wl	cg	ws	os	ss	in	rp	cr	wf				
YOLOv8n	96.6	89.3	94.6	71.8	74.7	70.1	19.4	10.0	38.2	92.1	65.7	3.01	846.9	8.1
YOLOv10b	94.2	89.1	93.8	81.1	64.5	62.0	30.3	26.1	55.1	93.9	69.0	20.43	197.1	98
YOLOv12l	99.5	82.8	91.4	85.1	78.2	69.4	26.6	13.8	62.1	96.3	70.5	26.35	144.0	88.6
Faster R-CNN	67.5	91.2	40.6	93.5	80.5	69.7	56.3	25.3	11.7	47.8	58.4	41.53	50.1	177.6
DEYOv1.5-E	99.4	92.3	92.5	78.6	68.7	66.8	34.0	34.5	26.9	98.8	69.3	58.62	74.7	173.9
YOLO-World	99.5	88.5	93.6	84.8	74.4	67.5	17.5	75.6	70.5	91.9	76.4	12.75	366.7	34.2
SCT-YOLO	99.5	96.7	97.1	85.3	79.3	86.4	39.3	50.2	52.0	98.1	78.4	5.66	260.3	15.2

## Data Availability

The datasets analyzed in this study are all included in the Supplementary Materials and are available from the corresponding author upon reasonable request.

## Supplementary Materials

The following supporting information can be downloaded at: https://www.mdpi.com/article/10.3390/s26123662/s1, Dataset S1: The SCT defect detection dataset used in this study.

## Abbreviations

The following abbreviations are used in this manuscript:

RPN	Region Proposal Network
YOLO	You Only Look Once
DSCA	Dual-Stream Collaborative Architecture
SCT	Shape–Color–Texture Multi-Dimensional Feature Images
MFM	Modulated Fusion Module
C2f_MSAA	Cross-Stage Multi-Scale Attention Aggregation Module
DC-SCConv	Dynamic Collaborative Spatial and Channel Reweighting Convolution
GWC	Grouped Weight-Wise Convolution
PWC	Point-Wise Convolution
AP	Average Precision
FPS	Frames Per Second
GFLOPs	Giga Floating-Point Operations Per Second
SGD	Stochastic Gradient Descent
IoU	Intersection over Union

## References

[1] Chen Y., Ding Y., Zhao F., Zhang E., Wu Z., Shao L. (2021). Surface Defect Detection Methods for Industrial Products: A Review. Appl. Sci..

[2] Neogi N., Mohanta D.K., Dutta P.K. (2014). Review of vision-based steel surface inspection systems. EURASIP J. Image Video Process..

[3] Tang B., Chen L., Sun W., Lin Z.k. (2023). Review of surface defect detection of steel products based on machine vision. IET Image Process..

[4] Wen X., Shan J., He Y., Song K. (2022). Steel Surface Defect Recognition: A Survey. Coatings.

[5] Yang J., Li S., Wang Z., Dong H., Wang J., Tang S. (2020). Using Deep Learning to Detect Defects in Manufacturing: A Comprehensive Survey and Current Challenges. Materials.

[6] Fang X., Luo Q., Zhou B., Li C., Tian L. (2020). Research Progress of Automated Visual Surface Defect Detection for Industrial Metal Planar Materials. Sensors.

[7] Ghalati M.K., Nunes A., Ferreira H., Serranho P., Bernardes R. (2022). Texture Analysis and Its Applications in Biomedical Imaging: A Survey. IEEE Rev. Biomed. Eng..

[8] Jing J., Liu S., Wang G., Zhang W., Sun C. (2022). Recent advances on image edge detection: A comprehensive review. Neurocomputing.

[9] Gu J., Wang Z., Kuen J., Ma L., Shahroudy A., Shuai B., Liu T., Wang X., Wang G., Cai J. (2018). Recent advances in convolutional neural networks. Pattern Recognit..

[10] Shin H.C., Roth H.R., Gao M., Lu L., Xu Z., Nogues I., Yao J., Mollura D., Summers R.M. (2016). Deep Convolutional Neural Networks for Computer-Aided Detection: CNN Architectures, Dataset Characteristics and Transfer Learning. IEEE Trans. Med. Imaging.

[11] Hochstein S., Ahissar M. (2002). View from the Top. Neuron.

[12] Wang S., Han K., Jin J. (2019). Review of image low-level feature extraction methods for content-based image retrieval. Sens. Rev..

[13] Zhu J.Y., Zhang Z., Zhang C., Wu J., Torralba A., Tenenbaum J.B., Freeman B. Visual Object Networks: Image Generation with Disentangled 3D Representations. Proceedings of the Neural Information Processing Systems.

[14] Prabhudesai M., Lal S., Patil D., Tung H.Y.F., Harley A.W., Fragkiadaki K. (2020). Disentangling 3D Prototypical Networks For Few-Shot Concept Learning. arXiv.

[15] Cant J.S., Large M.E., McCall L., Goodale M.A. (2008). Independent Processing of Form, Colour, and Texture in Object Perception. Perception.

[16] Geirhos R., Rubisch P., Michaelis C., Bethge M., Wichmann F., Brendel W. (2018). ImageNet-trained CNNs are biased towards texture; increasing shape bias improves accuracy and robustness. arXiv.

[17] Liang H., Sun X., Sun Y., Gao Y. (2017). Text feature extraction based on deep learning: A review. EURASIP J. Wirel. Commun. Netw..

[18] Ameri R., Hsu C.C., Band S.S. (2024). A systematic review of deep learning approaches for surface defect detection in industrial applications. Eng. Appl. Artif. Intell..

[19] Han H., Yang R., Li S., Hu R., Li X. (2023). SSGD: A smartphone screen glass dataset for defect detection. Proceedings of the ICASSP 2023–2023 IEEE International Conference on Acoustics, Speech and Signal Processing (ICASSP).

[20] Aryan P., Sampath S., Sohn H. (2018). An Overview of Non-Destructive Testing Methods for Integrated Circuit Packaging Inspection. Sensors.

[21] Ren S., He K., Girshick R., Sun J. (2017). Faster R-CNN: Towards Real-Time Object Detection with Region Proposal Networks. IEEE Trans. Pattern Anal. Mach. Intell..

[22] Jocher G., Chaurasia A., Qiu J. (2023). Ultralytics YOLOv8. https://docs.ultralytics.com/models/yolov8.

[23] Wang A., Chen H., Liu L., Chen K., Lin Z., Han J., Ding G. (2024). YOLOv10: Real-Time End-to-End Object Detection. arXiv.

[24] Cheng T., Song L., Ge Y., Liu W., Wang X., Shan Y. (2024). YOLO-World: Real-Time Open-Vocabulary Object Detection. arXiv.

[25] Khanam R., Hussain M. (2024). Yolov11: An overview of the key architectural enhancements. arXiv.

[26] Tian Y., Ye Q., Doermann D. (2025). YOLO12: Attention-Centric Real-Time Object Detectors. arXiv.

[27] Hu J., Shen L., Sun G. (2018). Squeeze-and-Excitation Networks. Proceedings of the 2018 IEEE/CVF Conference on Computer Vision and Pattern Recognition, Salt Lake City, UT, USA, 18–23 June 2018.

[28] Woo S., Park J., Lee J.Y., Kweon I.S., Ferrari V., Hebert M., Sminchisescu C., Weiss Y. (2018). CBAM: Convolutional Block Attention Module. Proceedings of the Computer Vision—ECCV 2018;Lecture Notes in Computer Science; Lecture Notes in Computer Science.

[29] Zhao Z., Xia C., Xie C., Li J. Complementary Trilateral Decoder for Fast and Accurate Salient Object Detection. Proceedings of the 29th ACM International Conference on Multimedia, Virtual Event China, 20–24 October 2021.

[30] Wang X., Girshick R., Gupta A., He K. (2018). Non-local Neural Networks. arXiv.

[31] Simonyan K., Zisserman A. (2014). Very Deep Convolutional Networks for Large-Scale Image Recognition. arXiv.

[32] He K., Zhang X., Ren S., Sun J. (2016). Deep Residual Learning for Image Recognition. Proceedings of the 2016 IEEE Conference on Computer Vision and Pattern Recognition (CVPR), Las Vegas, NV, USA, 27–30 June 2016.

[33] Han K., Wang Y., Tian Q., Guo J., Xu C., Xu C. (2020). GhostNet: More Features from Cheap Operations. arXiv.

[34] Zhang X., Zhou X., Lin M., Sun J. (2017). ShuffleNet: An Extremely Efficient Convolutional Neural Network for Mobile Devices. arXiv.

[35] Song G., Song K., Yan Y. (2020). Saliency detection for strip steel surface defects using multiple constraints and improved texture features. Opt. Lasers Eng..

[36] Rother C., Kolmogorov V., Blake A. (2004). “GrabCut”: Interactive foreground extraction using iterated graph cuts. ACM Trans. Graph..

[37] Ge Y., Xiao Y., Xu Z., Wang X., Itti L. (2022). Contributions of Shape, Texture, and Color in Visual Recognition. arXiv.

[38] Ranftl R., Bochkovskiy A., Koltun V. (2021). Vision Transformers for Dense Prediction. arXiv.

[39] Dosovitskiy A., Beyer L., Kolesnikov A., Weissenborn D., Zhai X., Unterthiner T., Dehghani M., Minderer M., Heigold G., Gelly S. (2021). An Image is Worth 16x16 Words: Transformers for Image Recognition at Scale. arXiv.

[40] Su J., Chen W.H., Yang J. (2016). On Relationship Between Time-Domain and Frequency-Domain Disturbance Observers and Its Applications. J. Dyn. Syst. Meas. Control.

[41] Vergeer M., Anstis S., Van Lier R. (2015). Flexible color perception depending on the shape and positioning of achromatic contours. Front. Psychol..

[42] Back J., Yoon S.E., Moon B. (2018). Feature Generation for Adaptive Gradient-Domain Path Tracing. Comput. Graph. Forum.

[43] Zhang Y., Zhou S., Li H. (2024). Depth Information Assisted Collaborative Mutual Promotion Network for Single Image Dehazing. Proceedings of the 2024 IEEE/CVF Conference on Computer Vision and Pattern Recognition (CVPR), Seattle, WA, USA, 16–22 June 2024.

[44] Mao M., Zhang R., Zheng H., Gao P., Ma T., Peng Y., Ding E., Zhang B., Han S. (2021). Dual-stream Network for Visual Recognition. arXiv.

[45] Liu M., Dan J., Lu Z., Yu Y., Li Y., Li X. (2024). CM-UNet: Hybrid CNN-Mamba UNet for Remote Sensing Image Semantic Segmentation. arXiv.

[46] Zhang K., Sun M., Han T.X., Yuan X., Guo L., Liu T. (2018). Residual Networks of Residual Networks: Multilevel Residual Networks. IEEE Trans. Circuits Syst. Video Technol..

[47] Li J., Wen Y., He L. (2023). SCConv: Spatial and Channel Reconstruction Convolution for Feature Redundancy. Proceedings of the 2023 IEEE/CVF Conference on Computer Vision and Pattern Recognition (CVPR), Vancouver, BC, Canada, 17–24 June 2023.

[48] Wan Y., Liao Z., Liu J., Song W., Ji H., Gao Z. (2023). Small object detection leveraging density-aware scale adaptation. Photogramm. Rec..

[49] Wu S., Yang J., Ma J., Zhang S., Hao T., Li M. (2025). SCDA-Net: Structure Completion and Density Awareness Network for LiDAR-Based 3D Object Detection. IEEE Robot. Autom. Lett..

[50] Wu Y., He K. (2018). Group Normalization. arXiv.

[51] Howard A.G., Zhu M., Chen B., Kalenichenko D., Wang W., Weyand T., Andreetto M., Adam H. (2017). MobileNets: Efficient Convolutional Neural Networks for Mobile Vision Applications. arXiv.

[52] Ouyang H. (2024). DEYO: DETR with YOLO for End-to-End Object Detection. arXiv.

[53] Lv X., Duan F., Jiang J.j., Fu X., Gan L. (2020). Deep Metallic Surface Defect Detection: The New Benchmark and Detection Network. Sensors.

