# Supplementary figures and images for: SCT-YOLO: A Dual-Stream Defect Detection Network Utilizing Computational Shape, Texture, and Color Features (part 2 of 2)
Source: Sensors (Basel). 2026 Jun 8;26(12):3662. doi: 10.3390/s26123662 (PMC13306621; doi:10.3390/s26123662)

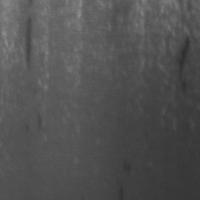

Supplement: Supplementary file 1 [file sensors-26-03662-s001.zip › NEU-900/images/train/In_284.jpg]

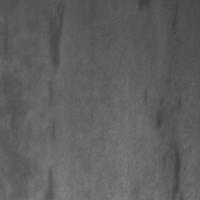

Supplement: Supplementary file 1 [file sensors-26-03662-s001.zip › NEU-900/images/train/In_62.jpg]

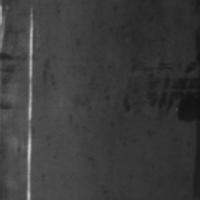

Supplement: Supplementary file 1 [file sensors-26-03662-s001.zip › NEU-900/images/train/Sc_228.jpg]

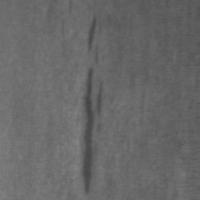

Supplement: Supplementary file 1 [file sensors-26-03662-s001.zip › NEU-900/images/train/In_172.jpg]

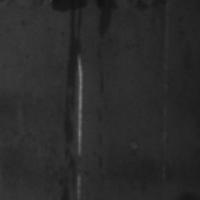

Supplement: Supplementary file 1 [file sensors-26-03662-s001.zip › NEU-900/images/train/Sc_229.jpg]

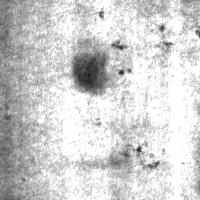

Supplement: Supplementary file 1 [file sensors-26-03662-s001.zip › NEU-900/images/train/Pa_39.jpg]

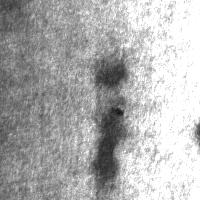

Supplement: Supplementary file 1 [file sensors-26-03662-s001.zip › NEU-900/images/train/Pa_156.jpg]

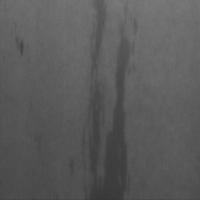

Supplement: Supplementary file 1 [file sensors-26-03662-s001.zip › NEU-900/images/train/In_276.jpg]

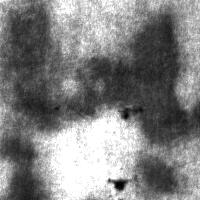

Supplement: Supplementary file 1 [file sensors-26-03662-s001.zip › NEU-900/images/train/Pa_26.jpg]

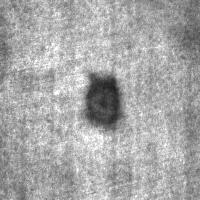

Supplement: Supplementary file 1 [file sensors-26-03662-s001.zip › NEU-900/images/train/Pa_94.jpg]

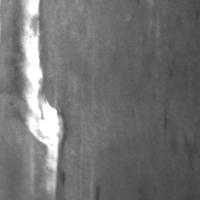

Supplement: Supplementary file 1 [file sensors-26-03662-s001.zip › NEU-900/images/train/Sc_122.jpg]

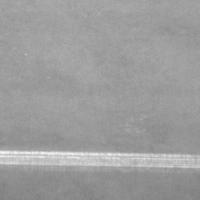

Supplement: Supplementary file 1 [file sensors-26-03662-s001.zip › NEU-900/images/train/Sc_46.jpg]

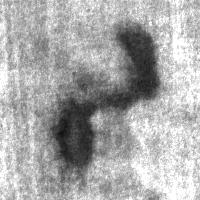

Supplement: Supplementary file 1 [file sensors-26-03662-s001.zip › NEU-900/images/train/Pa_75.jpg]

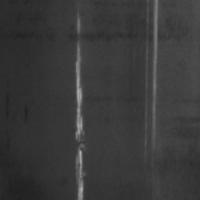

Supplement: Supplementary file 1 [file sensors-26-03662-s001.zip › NEU-900/images/train/Sc_199.jpg]

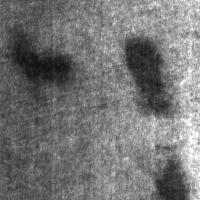

Supplement: Supplementary file 1 [file sensors-26-03662-s001.zip › NEU-900/images/train/Pa_279.jpg]

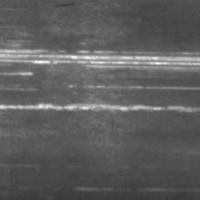

Supplement: Supplementary file 1 [file sensors-26-03662-s001.zip › NEU-900/images/train/Sc_279.jpg]

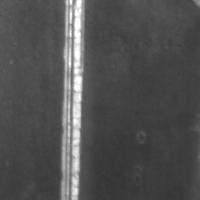

Supplement: Supplementary file 1 [file sensors-26-03662-s001.zip › NEU-900/images/train/Sc_182.jpg]

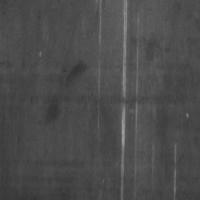

Supplement: Supplementary file 1 [file sensors-26-03662-s001.zip › NEU-900/images/train/Sc_208.jpg]

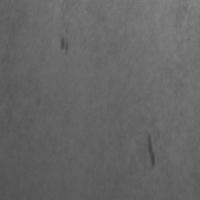

Supplement: Supplementary file 1 [file sensors-26-03662-s001.zip › NEU-900/images/train/In_142.jpg]

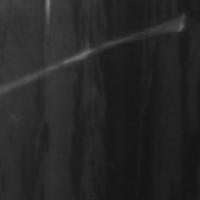

Supplement: Supplementary file 1 [file sensors-26-03662-s001.zip › NEU-900/images/train/Sc_126.jpg]

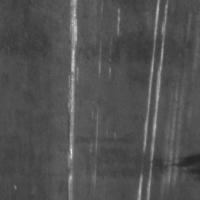

Supplement: Supplementary file 1 [file sensors-26-03662-s001.zip › NEU-900/images/train/Sc_211.jpg]

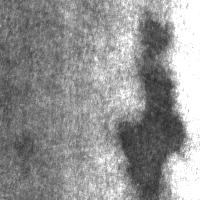

Supplement: Supplementary file 1 [file sensors-26-03662-s001.zip › NEU-900/images/train/Pa_293.jpg]

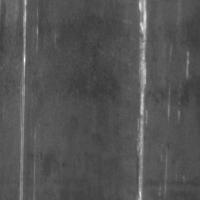

Supplement: Supplementary file 1 [file sensors-26-03662-s001.zip › NEU-900/images/train/Sc_212.jpg]

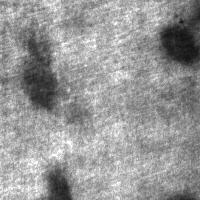

Supplement: Supplementary file 1 [file sensors-26-03662-s001.zip › NEU-900/images/train/Pa_68.jpg]

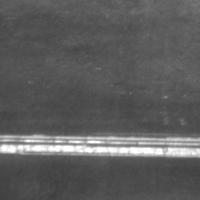

Supplement: Supplementary file 1 [file sensors-26-03662-s001.zip › NEU-900/images/train/Sc_258.jpg]

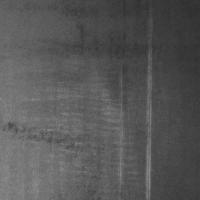

Supplement: Supplementary file 1 [file sensors-26-03662-s001.zip › NEU-900/images/train/Sc_84.jpg]

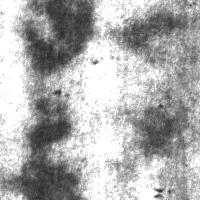

Supplement: Supplementary file 1 [file sensors-26-03662-s001.zip › NEU-900/images/train/Pa_243.jpg]

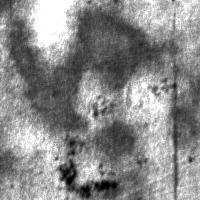

Supplement: Supplementary file 1 [file sensors-26-03662-s001.zip › NEU-900/images/train/Pa_199.jpg]

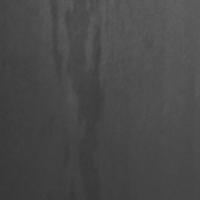

Supplement: Supplementary file 1 [file sensors-26-03662-s001.zip › NEU-900/images/train/In_217.jpg]

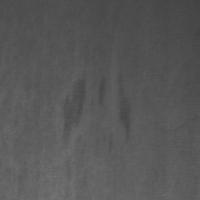

Supplement: Supplementary file 1 [file sensors-26-03662-s001.zip › NEU-900/images/train/In_218.jpg]

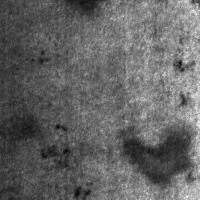

Supplement: Supplementary file 1 [file sensors-26-03662-s001.zip › NEU-900/images/train/Pa_240.jpg]

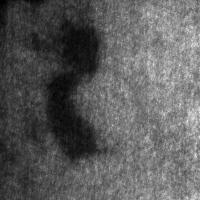

Supplement: Supplementary file 1 [file sensors-26-03662-s001.zip › NEU-900/images/train/Pa_300.jpg]

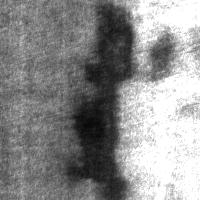

Supplement: Supplementary file 1 [file sensors-26-03662-s001.zip › NEU-900/images/train/Pa_46.jpg]

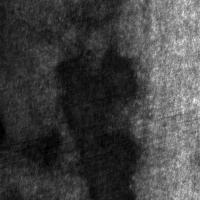

Supplement: Supplementary file 1 [file sensors-26-03662-s001.zip › NEU-900/images/train/Pa_208.jpg]

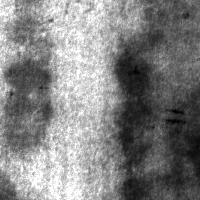

Supplement: Supplementary file 1 [file sensors-26-03662-s001.zip › NEU-900/images/train/Pa_269.jpg]

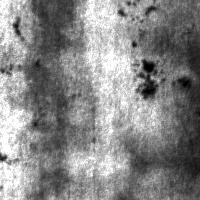

Supplement: Supplementary file 1 [file sensors-26-03662-s001.zip › NEU-900/images/train/Pa_190.jpg]

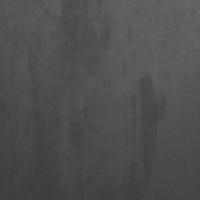

Supplement: Supplementary file 1 [file sensors-26-03662-s001.zip › NEU-900/images/train/In_34.jpg]

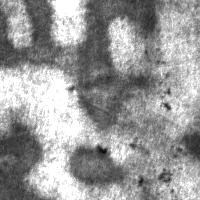

Supplement: Supplementary file 1 [file sensors-26-03662-s001.zip › NEU-900/images/train/Pa_231.jpg]

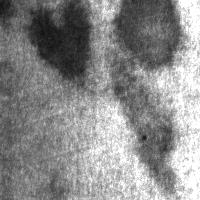

Supplement: Supplementary file 1 [file sensors-26-03662-s001.zip › NEU-900/images/train/Pa_296.jpg]

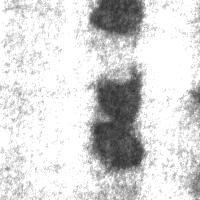

Supplement: Supplementary file 1 [file sensors-26-03662-s001.zip › NEU-900/images/train/Pa_270.jpg]

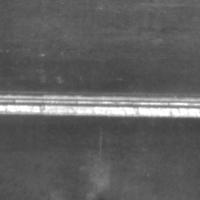

Supplement: Supplementary file 1 [file sensors-26-03662-s001.zip › NEU-900/images/train/Sc_259.jpg]

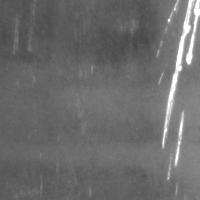

Supplement: Supplementary file 1 [file sensors-26-03662-s001.zip › NEU-900/images/train/Sc_8.jpg]

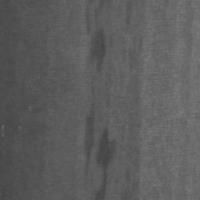

Supplement: Supplementary file 1 [file sensors-26-03662-s001.zip › NEU-900/images/train/In_54.jpg]

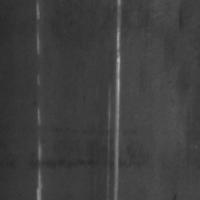

Supplement: Supplementary file 1 [file sensors-26-03662-s001.zip › NEU-900/images/train/Sc_198.jpg]

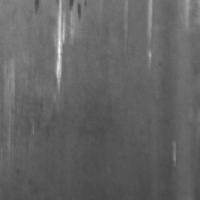

Supplement: Supplementary file 1 [file sensors-26-03662-s001.zip › NEU-900/images/train/Sc_124.jpg]

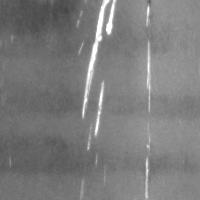

Supplement: Supplementary file 1 [file sensors-26-03662-s001.zip › NEU-900/images/train/Sc_10.jpg]

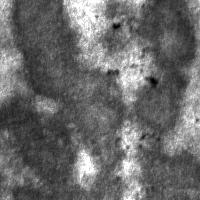

Supplement: Supplementary file 1 [file sensors-26-03662-s001.zip › NEU-900/images/train/Pa_215.jpg]

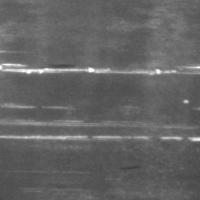

Supplement: Supplementary file 1 [file sensors-26-03662-s001.zip › NEU-900/images/train/Sc_270.jpg]

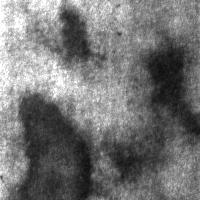

Supplement: Supplementary file 1 [file sensors-26-03662-s001.zip › NEU-900/images/train/Pa_115.jpg]

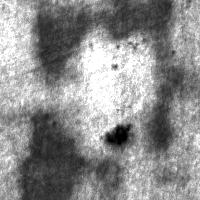

Supplement: Supplementary file 1 [file sensors-26-03662-s001.zip › NEU-900/images/train/Pa_202.jpg]

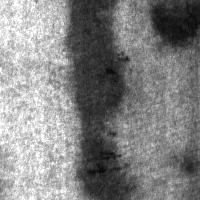

Supplement: Supplementary file 1 [file sensors-26-03662-s001.zip › NEU-900/images/train/Pa_285.jpg]

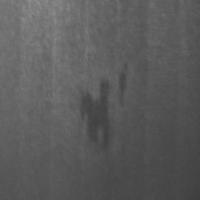

Supplement: Supplementary file 1 [file sensors-26-03662-s001.zip › NEU-900/images/train/In_71.jpg]

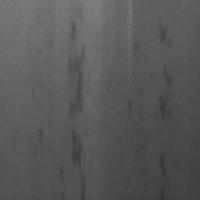

Supplement: Supplementary file 1 [file sensors-26-03662-s001.zip › NEU-900/images/train/In_275.jpg]

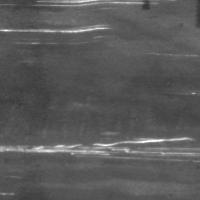

Supplement: Supplementary file 1 [file sensors-26-03662-s001.zip › NEU-900/images/train/Sc_238.jpg]

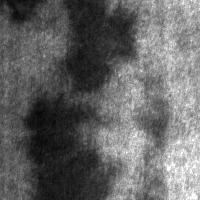

Supplement: Supplementary file 1 [file sensors-26-03662-s001.zip › NEU-900/images/train/Pa_138.jpg]

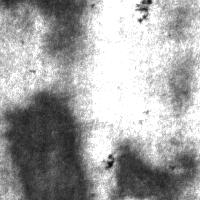

Supplement: Supplementary file 1 [file sensors-26-03662-s001.zip › NEU-900/images/train/Pa_28.jpg]

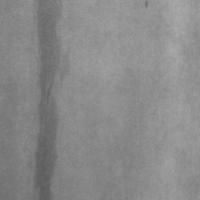

Supplement: Supplementary file 1 [file sensors-26-03662-s001.zip › NEU-900/images/train/In_277.jpg]

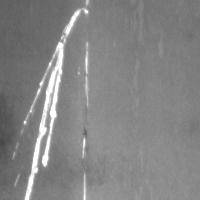

Supplement: Supplementary file 1 [file sensors-26-03662-s001.zip › NEU-900/images/train/Sc_7.jpg]

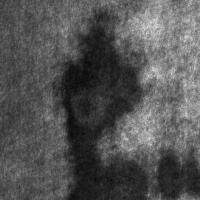

Supplement: Supplementary file 1 [file sensors-26-03662-s001.zip › NEU-900/images/train/Pa_123.jpg]

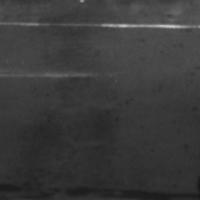

Supplement: Supplementary file 1 [file sensors-26-03662-s001.zip › NEU-900/images/train/Sc_245.jpg]

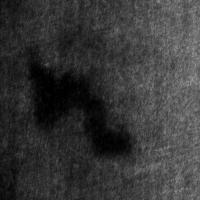

Supplement: Supplementary file 1 [file sensors-26-03662-s001.zip › NEU-900/images/train/Pa_153.jpg]

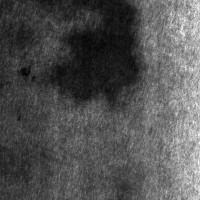

Supplement: Supplementary file 1 [file sensors-26-03662-s001.zip › NEU-900/images/train/Pa_170.jpg]

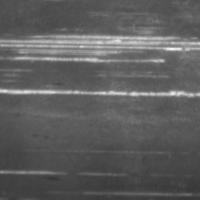

Supplement: Supplementary file 1 [file sensors-26-03662-s001.zip › NEU-900/images/train/Sc_280.jpg]

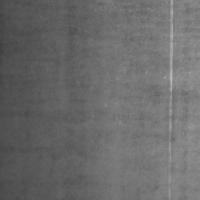

Supplement: Supplementary file 1 [file sensors-26-03662-s001.zip › NEU-900/images/train/Sc_73.jpg]

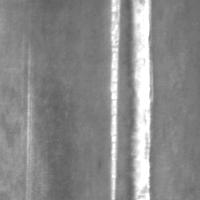

Supplement: Supplementary file 1 [file sensors-26-03662-s001.zip › NEU-900/images/train/Sc_117.jpg]

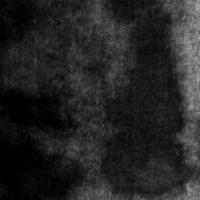

Supplement: Supplementary file 1 [file sensors-26-03662-s001.zip › NEU-900/images/train/Pa_218.jpg]

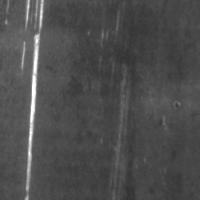

Supplement: Supplementary file 1 [file sensors-26-03662-s001.zip › NEU-900/images/train/Sc_191.jpg]

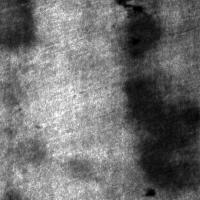

Supplement: Supplementary file 1 [file sensors-26-03662-s001.zip › NEU-900/images/train/Pa_71.jpg]

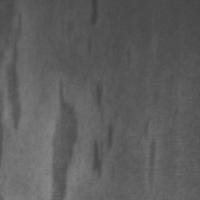

Supplement: Supplementary file 1 [file sensors-26-03662-s001.zip › NEU-900/images/train/In_114.jpg]

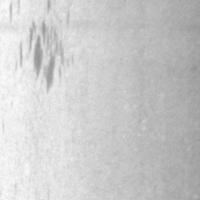

Supplement: Supplementary file 1 [file sensors-26-03662-s001.zip › NEU-900/images/train/In_236.jpg]

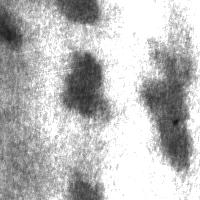

Supplement: Supplementary file 1 [file sensors-26-03662-s001.zip › NEU-900/images/train/Pa_292.jpg]

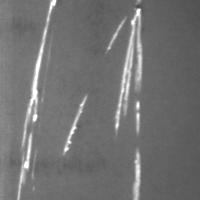

Supplement: Supplementary file 1 [file sensors-26-03662-s001.zip › NEU-900/images/train/Sc_27.jpg]

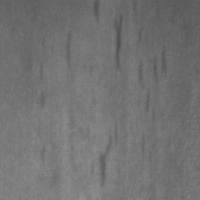

Supplement: Supplementary file 1 [file sensors-26-03662-s001.zip › NEU-900/images/train/In_88.jpg]

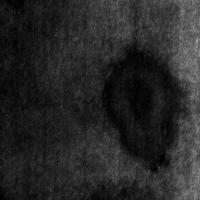

Supplement: Supplementary file 1 [file sensors-26-03662-s001.zip › NEU-900/images/train/Pa_164.jpg]

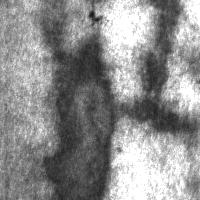

Supplement: Supplementary file 1 [file sensors-26-03662-s001.zip › NEU-900/images/train/Pa_183.jpg]

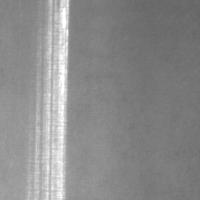

Supplement: Supplementary file 1 [file sensors-26-03662-s001.zip › NEU-900/images/train/Sc_50.jpg]

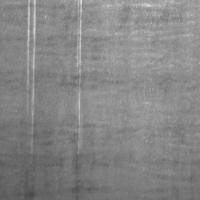

Supplement: Supplementary file 1 [file sensors-26-03662-s001.zip › NEU-900/images/train/Sc_83.jpg]

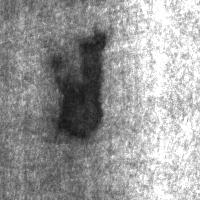

Supplement: Supplementary file 1 [file sensors-26-03662-s001.zip › NEU-900/images/train/Pa_272.jpg]

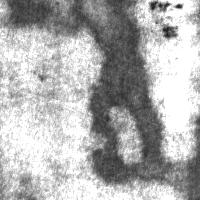

Supplement: Supplementary file 1 [file sensors-26-03662-s001.zip › NEU-900/images/train/Pa_229.jpg]

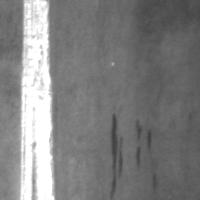

Supplement: Supplementary file 1 [file sensors-26-03662-s001.zip › NEU-900/images/train/Sc_139.jpg]

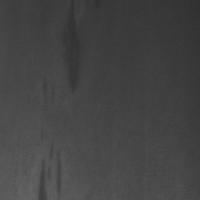

Supplement: Supplementary file 1 [file sensors-26-03662-s001.zip › NEU-900/images/train/In_82.jpg]

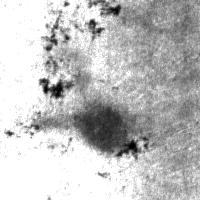

Supplement: Supplementary file 1 [file sensors-26-03662-s001.zip › NEU-900/images/train/Pa_44.jpg]

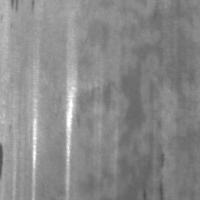

Supplement: Supplementary file 1 [file sensors-26-03662-s001.zip › NEU-900/images/train/Sc_93.jpg]

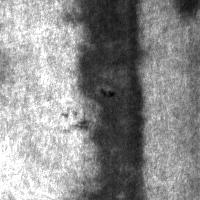

Supplement: Supplementary file 1 [file sensors-26-03662-s001.zip › NEU-900/images/train/Pa_287.jpg]

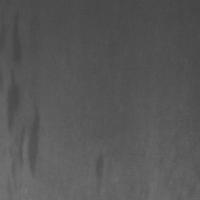

Supplement: Supplementary file 1 [file sensors-26-03662-s001.zip › NEU-900/images/train/In_14.jpg]

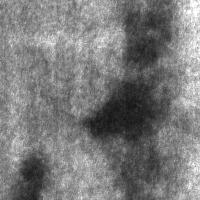

Supplement: Supplementary file 1 [file sensors-26-03662-s001.zip › NEU-900/images/train/Pa_12.jpg]

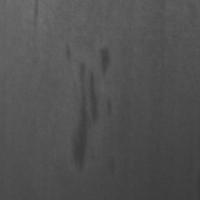

Supplement: Supplementary file 1 [file sensors-26-03662-s001.zip › NEU-900/images/train/In_128.jpg]

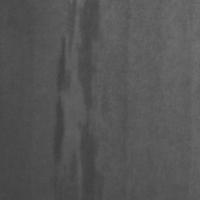

Supplement: Supplementary file 1 [file sensors-26-03662-s001.zip › NEU-900/images/train/In_201.jpg]

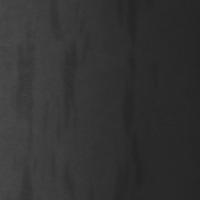

Supplement: Supplementary file 1 [file sensors-26-03662-s001.zip › NEU-900/images/train/In_39.jpg]

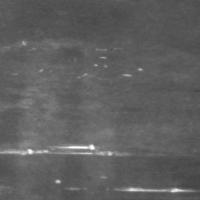

Supplement: Supplementary file 1 [file sensors-26-03662-s001.zip › NEU-900/images/train/Sc_267.jpg]

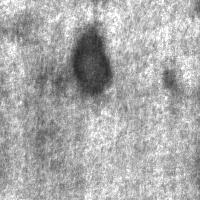

Supplement: Supplementary file 1 [file sensors-26-03662-s001.zip › NEU-900/images/train/Pa_135.jpg]

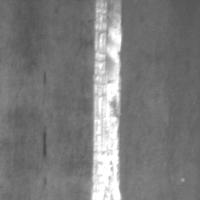

Supplement: Supplementary file 1 [file sensors-26-03662-s001.zip › NEU-900/images/train/Sc_137.jpg]

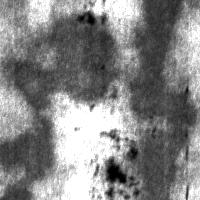

Supplement: Supplementary file 1 [file sensors-26-03662-s001.zip › NEU-900/images/train/Pa_175.jpg]

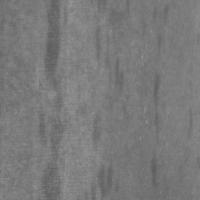

Supplement: Supplementary file 1 [file sensors-26-03662-s001.zip › NEU-900/images/train/In_28.jpg]

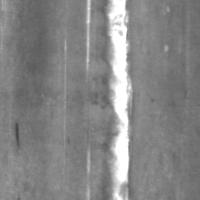

Supplement: Supplementary file 1 [file sensors-26-03662-s001.zip › NEU-900/images/train/Sc_120.jpg]

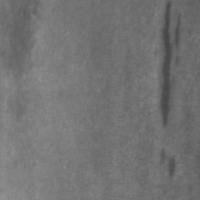

Supplement: Supplementary file 1 [file sensors-26-03662-s001.zip › NEU-900/images/train/In_173.jpg]

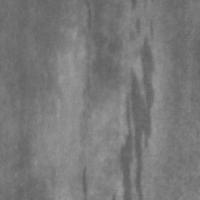

Supplement: Supplementary file 1 [file sensors-26-03662-s001.zip › NEU-900/images/train/In_121.jpg]

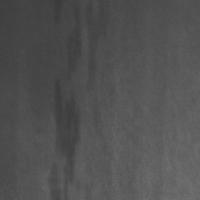

Supplement: Supplementary file 1 [file sensors-26-03662-s001.zip › NEU-900/images/train/In_48.jpg]
