# Supplementary figures and images for: SCT-YOLO: A Dual-Stream Defect Detection Network Utilizing Computational Shape, Texture, and Color Features (part 1 of 2)
Source: Sensors (Basel). 2026 Jun 8;26(12):3662. doi: 10.3390/s26123662 (PMC13306621; doi:10.3390/s26123662)

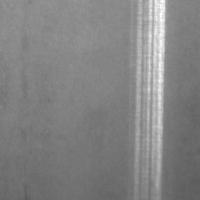

Supplement: Supplementary file 1 [file sensors-26-03662-s001.zip › NEU-900/images/train/Sc_56.jpg]

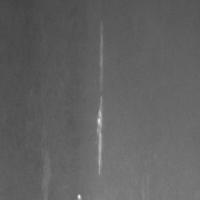

Supplement: Supplementary file 1 [file sensors-26-03662-s001.zip › NEU-900/images/train/Sc_2.jpg]

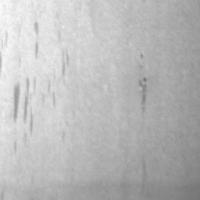

Supplement: Supplementary file 1 [file sensors-26-03662-s001.zip › NEU-900/images/train/In_243.jpg]

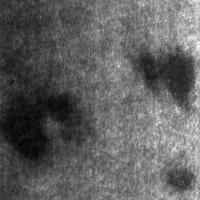

Supplement: Supplementary file 1 [file sensors-26-03662-s001.zip › NEU-900/images/train/Pa_298.jpg]

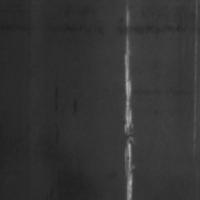

Supplement: Supplementary file 1 [file sensors-26-03662-s001.zip › NEU-900/images/train/Sc_200.jpg]

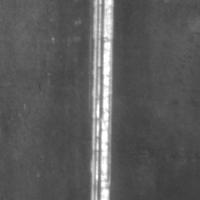

Supplement: Supplementary file 1 [file sensors-26-03662-s001.zip › NEU-900/images/train/Sc_181.jpg]

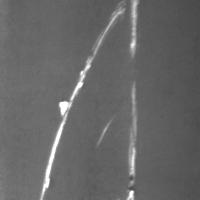

Supplement: Supplementary file 1 [file sensors-26-03662-s001.zip › NEU-900/images/train/Sc_23.jpg]

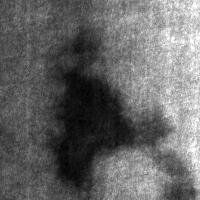

Supplement: Supplementary file 1 [file sensors-26-03662-s001.zip › NEU-900/images/train/Pa_31.jpg]

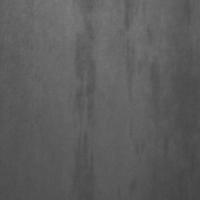

Supplement: Supplementary file 1 [file sensors-26-03662-s001.zip › NEU-900/images/train/In_33.jpg]

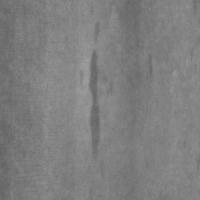

Supplement: Supplementary file 1 [file sensors-26-03662-s001.zip › NEU-900/images/train/In_180.jpg]

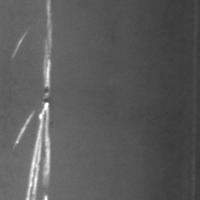

Supplement: Supplementary file 1 [file sensors-26-03662-s001.zip › NEU-900/images/train/Sc_24.jpg]

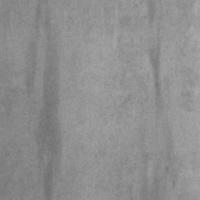

Supplement: Supplementary file 1 [file sensors-26-03662-s001.zip › NEU-900/images/train/In_261.jpg]

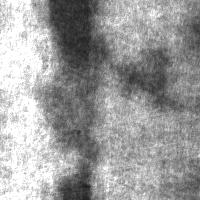

Supplement: Supplementary file 1 [file sensors-26-03662-s001.zip › NEU-900/images/train/Pa_18.jpg]

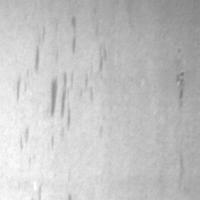

Supplement: Supplementary file 1 [file sensors-26-03662-s001.zip › NEU-900/images/train/In_233.jpg]

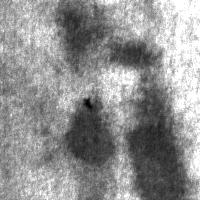

Supplement: Supplementary file 1 [file sensors-26-03662-s001.zip › NEU-900/images/train/Pa_189.jpg]

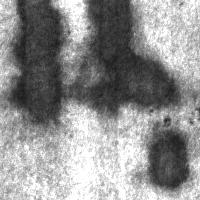

Supplement: Supplementary file 1 [file sensors-26-03662-s001.zip › NEU-900/images/train/Pa_108.jpg]

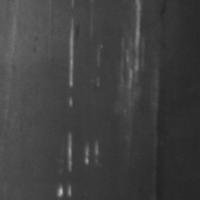

Supplement: Supplementary file 1 [file sensors-26-03662-s001.zip › NEU-900/images/train/Sc_168.jpg]

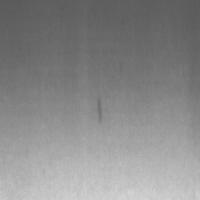

Supplement: Supplementary file 1 [file sensors-26-03662-s001.zip › NEU-900/images/train/In_228.jpg]

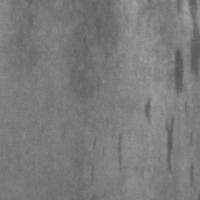

Supplement: Supplementary file 1 [file sensors-26-03662-s001.zip › NEU-900/images/train/In_130.jpg]

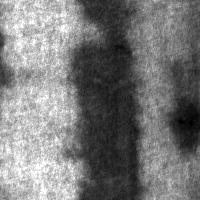

Supplement: Supplementary file 1 [file sensors-26-03662-s001.zip › NEU-900/images/train/Pa_286.jpg]

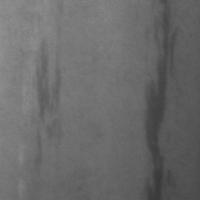

Supplement: Supplementary file 1 [file sensors-26-03662-s001.zip › NEU-900/images/train/In_266.jpg]

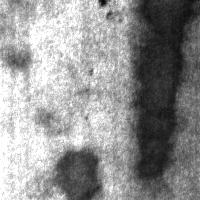

Supplement: Supplementary file 1 [file sensors-26-03662-s001.zip › NEU-900/images/train/Pa_111.jpg]

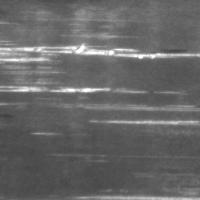

Supplement: Supplementary file 1 [file sensors-26-03662-s001.zip › NEU-900/images/train/Sc_271.jpg]

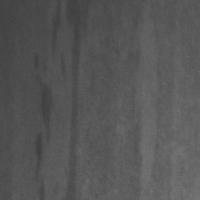

Supplement: Supplementary file 1 [file sensors-26-03662-s001.zip › NEU-900/images/train/In_181.jpg]

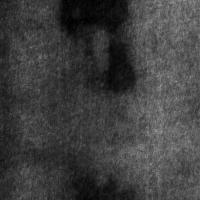

Supplement: Supplementary file 1 [file sensors-26-03662-s001.zip › NEU-900/images/train/Pa_133.jpg]

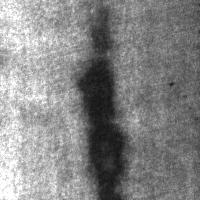

Supplement: Supplementary file 1 [file sensors-26-03662-s001.zip › NEU-900/images/train/Pa_83.jpg]

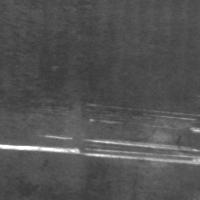

Supplement: Supplementary file 1 [file sensors-26-03662-s001.zip › NEU-900/images/train/Sc_253.jpg]

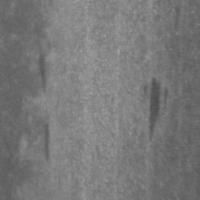

Supplement: Supplementary file 1 [file sensors-26-03662-s001.zip › NEU-900/images/train/In_69.jpg]

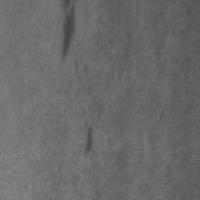

Supplement: Supplementary file 1 [file sensors-26-03662-s001.zip › NEU-900/images/train/In_149.jpg]

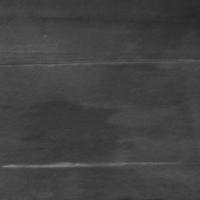

Supplement: Supplementary file 1 [file sensors-26-03662-s001.zip › NEU-900/images/train/Sc_246.jpg]

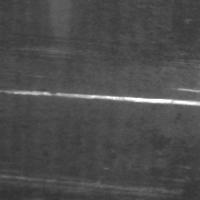

Supplement: Supplementary file 1 [file sensors-26-03662-s001.zip › NEU-900/images/train/Sc_250.jpg]

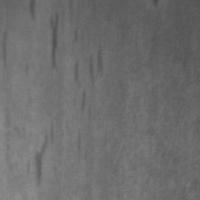

Supplement: Supplementary file 1 [file sensors-26-03662-s001.zip › NEU-900/images/train/In_91.jpg]

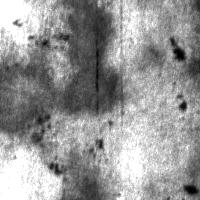

Supplement: Supplementary file 1 [file sensors-26-03662-s001.zip › NEU-900/images/train/Pa_187.jpg]

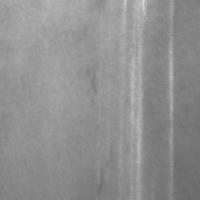

Supplement: Supplementary file 1 [file sensors-26-03662-s001.zip › NEU-900/images/train/Sc_70.jpg]

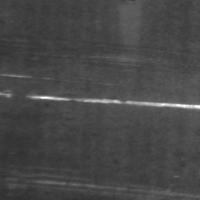

Supplement: Supplementary file 1 [file sensors-26-03662-s001.zip › NEU-900/images/train/Sc_249.jpg]

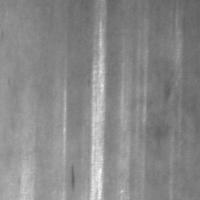

Supplement: Supplementary file 1 [file sensors-26-03662-s001.zip › NEU-900/images/train/Sc_91.jpg]

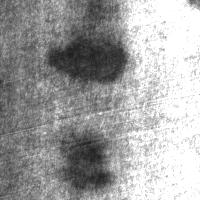

Supplement: Supplementary file 1 [file sensors-26-03662-s001.zip › NEU-900/images/train/Pa_254.jpg]

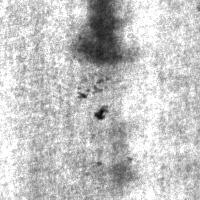

Supplement: Supplementary file 1 [file sensors-26-03662-s001.zip › NEU-900/images/train/Pa_61.jpg]

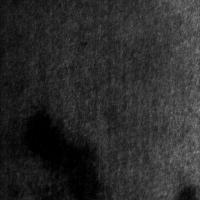

Supplement: Supplementary file 1 [file sensors-26-03662-s001.zip › NEU-900/images/train/Pa_162.jpg]

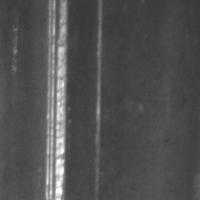

Supplement: Supplementary file 1 [file sensors-26-03662-s001.zip › NEU-900/images/train/Sc_177.jpg]

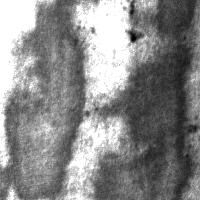

Supplement: Supplementary file 1 [file sensors-26-03662-s001.zip › NEU-900/images/train/Pa_158.jpg]

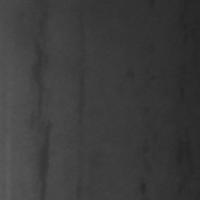

Supplement: Supplementary file 1 [file sensors-26-03662-s001.zip › NEU-900/images/train/In_271.jpg]

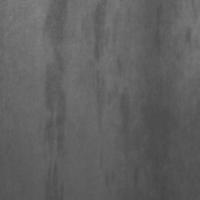

Supplement: Supplementary file 1 [file sensors-26-03662-s001.zip › NEU-900/images/train/In_32.jpg]

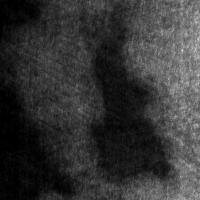

Supplement: Supplementary file 1 [file sensors-26-03662-s001.zip › NEU-900/images/train/Pa_299.jpg]

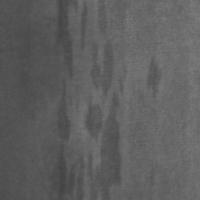

Supplement: Supplementary file 1 [file sensors-26-03662-s001.zip › NEU-900/images/train/In_105.jpg]

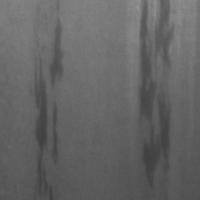

Supplement: Supplementary file 1 [file sensors-26-03662-s001.zip › NEU-900/images/train/In_264.jpg]

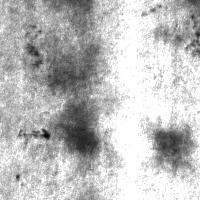

Supplement: Supplementary file 1 [file sensors-26-03662-s001.zip › NEU-900/images/train/Pa_34.jpg]

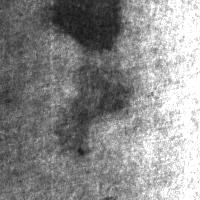

Supplement: Supplementary file 1 [file sensors-26-03662-s001.zip › NEU-900/images/train/Pa_238.jpg]

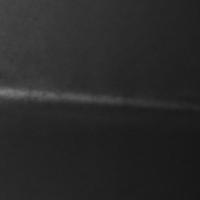

Supplement: Supplementary file 1 [file sensors-26-03662-s001.zip › NEU-900/images/train/Sc_103.jpg]

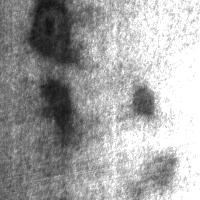

Supplement: Supplementary file 1 [file sensors-26-03662-s001.zip › NEU-900/images/train/Pa_154.jpg]

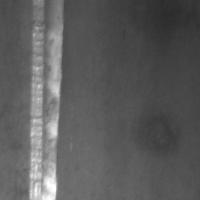

Supplement: Supplementary file 1 [file sensors-26-03662-s001.zip › NEU-900/images/train/Sc_134.jpg]

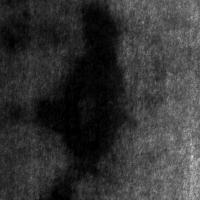

Supplement: Supplementary file 1 [file sensors-26-03662-s001.zip › NEU-900/images/train/Pa_143.jpg]

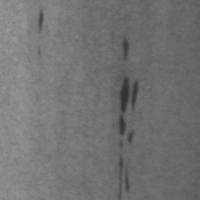

Supplement: Supplementary file 1 [file sensors-26-03662-s001.zip › NEU-900/images/train/In_1.jpg]

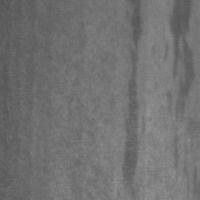

Supplement: Supplementary file 1 [file sensors-26-03662-s001.zip › NEU-900/images/train/In_16.jpg]

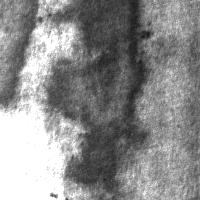

Supplement: Supplementary file 1 [file sensors-26-03662-s001.zip › NEU-900/images/train/Pa_157.jpg]

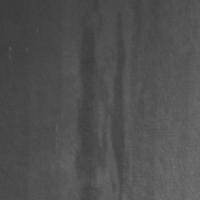

Supplement: Supplementary file 1 [file sensors-26-03662-s001.zip › NEU-900/images/train/In_106.jpg]

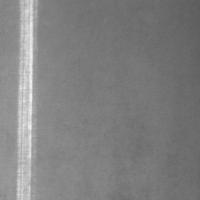

Supplement: Supplementary file 1 [file sensors-26-03662-s001.zip › NEU-900/images/train/Sc_39.jpg]

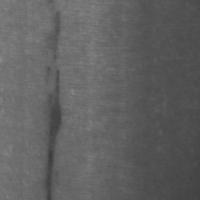

Supplement: Supplementary file 1 [file sensors-26-03662-s001.zip › NEU-900/images/train/In_286.jpg]

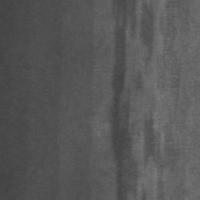

Supplement: Supplementary file 1 [file sensors-26-03662-s001.zip › NEU-900/images/train/In_101.jpg]

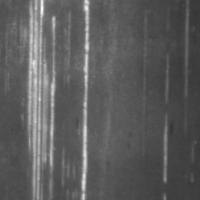

Supplement: Supplementary file 1 [file sensors-26-03662-s001.zip › NEU-900/images/train/Sc_166.jpg]

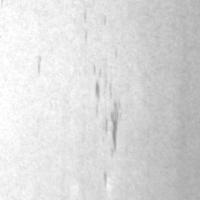

Supplement: Supplementary file 1 [file sensors-26-03662-s001.zip › NEU-900/images/train/In_238.jpg]

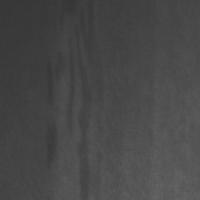

Supplement: Supplementary file 1 [file sensors-26-03662-s001.zip › NEU-900/images/train/In_159.jpg]

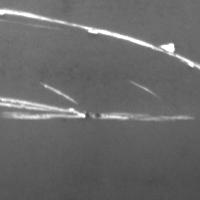

Supplement: Supplementary file 1 [file sensors-26-03662-s001.zip › NEU-900/images/train/Sc_294.jpg]

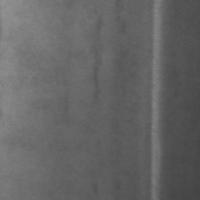

Supplement: Supplementary file 1 [file sensors-26-03662-s001.zip › NEU-900/images/train/Sc_69.jpg]

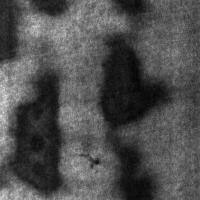

Supplement: Supplementary file 1 [file sensors-26-03662-s001.zip › NEU-900/images/train/Pa_92.jpg]

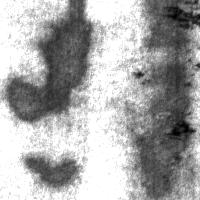

Supplement: Supplementary file 1 [file sensors-26-03662-s001.zip › NEU-900/images/train/Pa_267.jpg]

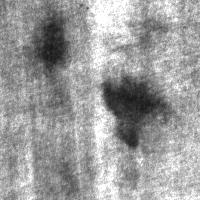

Supplement: Supplementary file 1 [file sensors-26-03662-s001.zip › NEU-900/images/train/Pa_66.jpg]

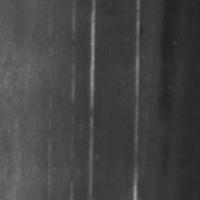

Supplement: Supplementary file 1 [file sensors-26-03662-s001.zip › NEU-900/images/train/Sc_167.jpg]

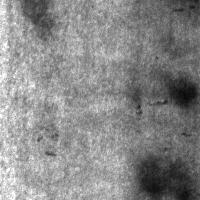

Supplement: Supplementary file 1 [file sensors-26-03662-s001.zip › NEU-900/images/train/Pa_77.jpg]

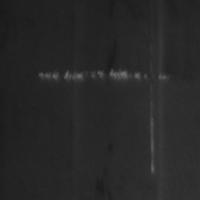

Supplement: Supplementary file 1 [file sensors-26-03662-s001.zip › NEU-900/images/train/Sc_232.jpg]

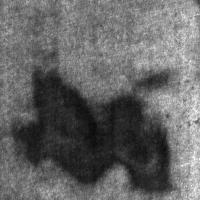

Supplement: Supplementary file 1 [file sensors-26-03662-s001.zip › NEU-900/images/train/Pa_98.jpg]

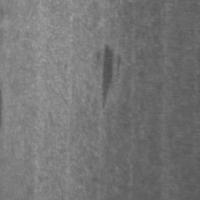

Supplement: Supplementary file 1 [file sensors-26-03662-s001.zip › NEU-900/images/train/In_72.jpg]

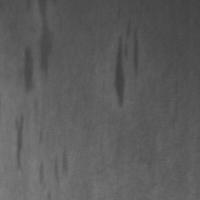

Supplement: Supplementary file 1 [file sensors-26-03662-s001.zip › NEU-900/images/train/In_86.jpg]

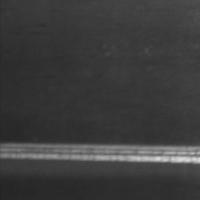

Supplement: Supplementary file 1 [file sensors-26-03662-s001.zip › NEU-900/images/train/Sc_255.jpg]

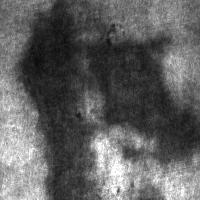

Supplement: Supplementary file 1 [file sensors-26-03662-s001.zip › NEU-900/images/train/Pa_6.jpg]

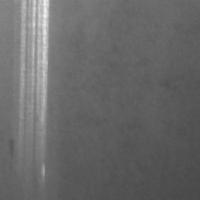

Supplement: Supplementary file 1 [file sensors-26-03662-s001.zip › NEU-900/images/train/Sc_58.jpg]

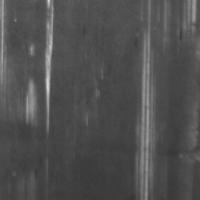

Supplement: Supplementary file 1 [file sensors-26-03662-s001.zip › NEU-900/images/train/Sc_161.jpg]

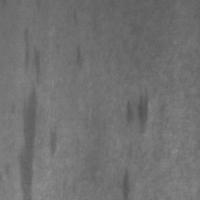

Supplement: Supplementary file 1 [file sensors-26-03662-s001.zip › NEU-900/images/train/In_17.jpg]

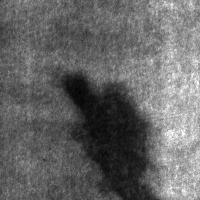

Supplement: Supplementary file 1 [file sensors-26-03662-s001.zip › NEU-900/images/train/Pa_70.jpg]

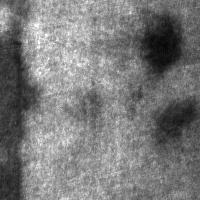

Supplement: Supplementary file 1 [file sensors-26-03662-s001.zip › NEU-900/images/train/Pa_24.jpg]

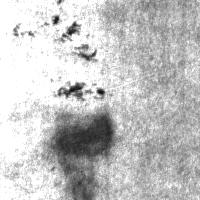

Supplement: Supplementary file 1 [file sensors-26-03662-s001.zip › NEU-900/images/train/Pa_37.jpg]

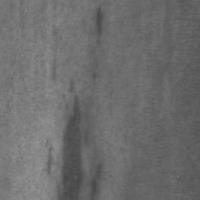

Supplement: Supplementary file 1 [file sensors-26-03662-s001.zip › NEU-900/images/train/In_208.jpg]

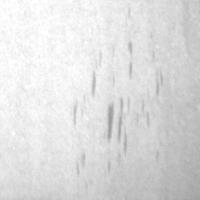

Supplement: Supplementary file 1 [file sensors-26-03662-s001.zip › NEU-900/images/train/In_242.jpg]

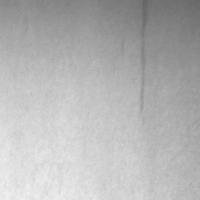

Supplement: Supplementary file 1 [file sensors-26-03662-s001.zip › NEU-900/images/train/In_253.jpg]

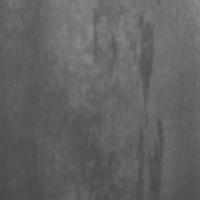

Supplement: Supplementary file 1 [file sensors-26-03662-s001.zip › NEU-900/images/train/In_155.jpg]

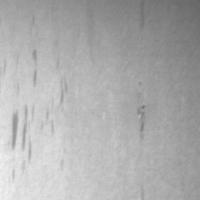

Supplement: Supplementary file 1 [file sensors-26-03662-s001.zip › NEU-900/images/train/In_232.jpg]

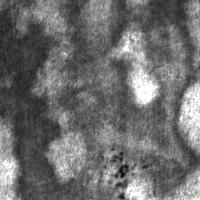

Supplement: Supplementary file 1 [file sensors-26-03662-s001.zip › NEU-900/images/train/Pa_210.jpg]

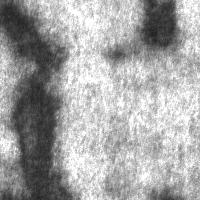

Supplement: Supplementary file 1 [file sensors-26-03662-s001.zip › NEU-900/images/train/Pa_146.jpg]

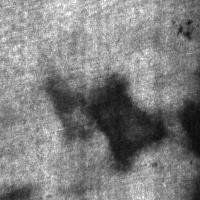

Supplement: Supplementary file 1 [file sensors-26-03662-s001.zip › NEU-900/images/train/Pa_95.jpg]

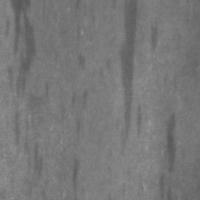

Supplement: Supplementary file 1 [file sensors-26-03662-s001.zip › NEU-900/images/train/In_18.jpg]

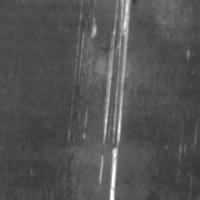

Supplement: Supplementary file 1 [file sensors-26-03662-s001.zip › NEU-900/images/train/Sc_188.jpg]

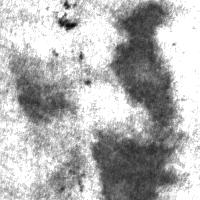

Supplement: Supplementary file 1 [file sensors-26-03662-s001.zip › NEU-900/images/train/Pa_235.jpg]

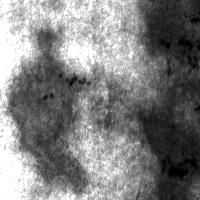

Supplement: Supplementary file 1 [file sensors-26-03662-s001.zip › NEU-900/images/train/Pa_297.jpg]

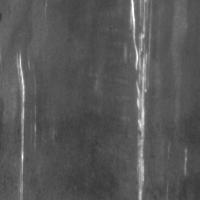

Supplement: Supplementary file 1 [file sensors-26-03662-s001.zip › NEU-900/images/train/Sc_214.jpg]

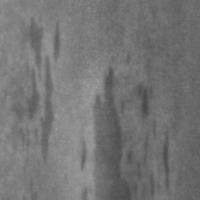

Supplement: Supplementary file 1 [file sensors-26-03662-s001.zip › NEU-900/images/train/In_151.jpg]

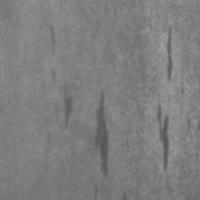

Supplement: Supplementary file 1 [file sensors-26-03662-s001.zip › NEU-900/images/train/In_93.jpg]

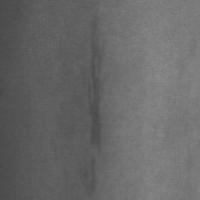

Supplement: Supplementary file 1 [file sensors-26-03662-s001.zip › NEU-900/images/train/In_263.jpg]

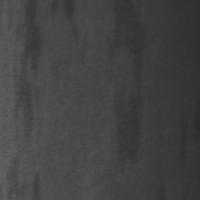

Supplement: Supplementary file 1 [file sensors-26-03662-s001.zip › NEU-900/images/train/In_25.jpg]

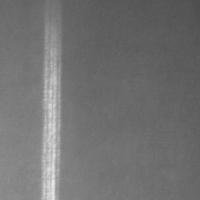

Supplement: Supplementary file 1 [file sensors-26-03662-s001.zip › NEU-900/images/train/Sc_36.jpg]

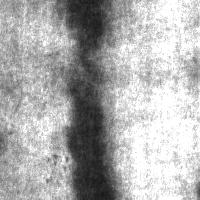

Supplement: Supplementary file 1 [file sensors-26-03662-s001.zip › NEU-900/images/train/Pa_15.jpg]
